# Active CNS delivery of oxycodone in healthy and endotoxemic pigs

**DOI:** 10.1186/s12987-024-00583-z

**Published:** 2024-10-23

**Authors:** Frida Bällgren, Tilda Bergfast, Aghavni Ginosyan, Jessica Mahajan, Miklós Lipcsey, Margareta Hammarlund-Udenaes, Stina Syvänen, Irena Loryan

**Affiliations:** 1 Translational Pharmacokinetics/Pharmacodynamics Group (tPKPD), Department of Pharmacy, Uppsala University, Husargatan 3, 752 37 Uppsala, Sweden; 2 Present Address: School of Applied Sciences, Abertay University, Bell Street, Dundee, DD1 1HG Scotland, UK; 3 Hedenstierna Laboratory, Department of Surgical Sciences, Uppsala University, Akademiska Sjukhuset, 751 85 Uppsala, Sweden; 4 Molecular Geriatrics, Department of Public Health and Caring Sciences, Uppsala University, Rudbecklaboratoriet, Dag Hammarskjölds Väg 20, 751 85 Uppsala, Sweden

**Keywords:** Oxycodone, Microdialysis, Blood–brain barrier, Blood-cerebrospinal fluid barrier, Brain interstitial fluid, Cerebrospinal fluid, Proton-coupled organic cation antiporter, Porcine, Lipopolysaccharide, Endotoxemia

## Abstract

**Background:**

The primary objective of this study was to advance our understanding of active drug uptake at brain barriers in higher species than rodents, by examining oxycodone brain concentrations in pigs.

**Methods:**

This was investigated by a microdialysis study in healthy and endotoxemic conditions to increase the understanding of inter-species translation of putative proton-coupled organic cation (H^+^/OC) antiporter-mediated central nervous system (CNS) drug delivery in health and pathology, and facilitate the extrapolation to humans for improved CNS drug treatment in patients. Additionally, we sought to evaluate the efficacy of lumbar cerebrospinal fluid (CSF) exposure readout as a proxy for brain unbound interstitial fluid (ISF) concentrations. By simultaneously monitoring unbound concentrations in blood, the frontal cortical area, the lateral ventricle (LV), and the lumbar intrathecal space in healthy and lipopolysaccharide (LPS)-induced inflammation states within the same animal, we achieved exceptional spatiotemporal resolution in mapping oxycodone transport across CNS barriers.

**Results:**

Our findings provide novel evidence of higher unbound oxycodone concentrations in brain ISF compared to blood, yielding an unbound brain-to-plasma concentration ratio (K_p,uu,brain_) of 2.5. This supports the hypothesis of the presence of the H^+^/OC antiporter system at the blood–brain barrier (BBB) in pigs. Despite significant physiological changes, reflected in pig Sequential Organ Failure Assessment, pSOFA scores, oxycodone blood concentrations and its active net uptake across the BBB remained nearly unchanged during three hours of i.v. infusion of 4 µg/kg/h LPS from *Escherichia coli* (O111:B4). Mean K_p,uu,LV_ values indicated active uptake also at the blood-CSF barrier in healthy and endotoxemic pigs. Lumbar CSF concentrations showed minimal inter-individual variability during the experiment, with a mean K_p,uu,lumbarCSF_ of 1.5. LPS challenge caused a slight decrease in K_p,uu,LV_, while K_p,uu,lumbarCSF_ remained unaffected.

**Conclusions:**

This study enhances our understanding of oxycodone pharmacokinetics and CNS drug delivery in both healthy and inflamed conditions, providing crucial insights for translating these findings to clinical settings.

**Supplementary Information:**

The online version contains supplementary material available at 10.1186/s12987-024-00583-z.

## Introduction

Successful treatment of brain diseases and the development of novel neurotherapeutics targeting the brain requires a thorough characterization of brain drug disposition in both healthy and pathological states in higher species. Notably, understanding of brain drug disposition of marketed organic cation drugs presents distinct opportunities due to their predominant ionization at physiological pH, which limits passive diffusion and necessitates interactions with membrane transporters to facilitate brain entry. Brain barriers, primarily, the blood–brain (BBB) and blood-cerebrospinal fluid (BCSFB) barriers, selectively regulate the transfer of drug molecules between the blood and the brain and employ various organic cation transporters to mediate this process [1]. Among those is the pyrilamine-sensitive proton-coupled organic cation (H^+^/OC) antiporter system, which has also emerged as a promising target for central nervous system (CNS) drug delivery [2, 3]. This system has been associated with the active net brain uptake of several drugs, including oxycodone [4, 5], diphenhydramine [6, 7], and pyrilamine [8, 9]. The widely used opioid analgesic oxycodone is a model drug to study the H^+^/OC antiporter system. It has been systematically investigated with a demonstration of its active uptake across both the BBB and the BCSFB in healthy and pathological conditions in rodents [5, 10–12]. Despite these advances, the presence and functionality of this active transport mechanism in higher species, such as pigs, remain mainly unexplored. Given the increasing relevance of pigs as a biomedical translational model [13–16], investigating the H^+^/OC antiporter system using oxycodone in these animals in healthy and pathological conditions is crucial for advancing our understanding of brain drug disposition and optimizing neurotherapeutic strategies.

Inflammation, prevalent in numerous infectious and non-infectious disorders, may alter BBB function [17, 18]. In this study, we utilized endotoxin, a lipopolysaccharide (LPS) component of the outer cell membrane of Gram-negative bacteria, as a well-characterized pathogen-associated molecular pattern, to induce inflammation. Clinical studies have demonstrated that LPS is pivotal in triggering a cascade of events leading to clinical and laboratory manifestations of a disbalance between systemic inflammatory response (SIRS) and compensatory anti-inflammatory response syndromes, sepsis, and septic shock [19–21]. Several experimental studies have shown dose- and time-dependent induction of SIRS and a septic-like state in pigs using LPS [22–26]. The Sequential Organ Failure Assessment (SOFA) score is a valuable tool for the diagnosis and monitoring of sepsis in critically ill patients [27, 28]. Lately, it has also been adapted for sepsis-mimicking pig models as pSOFA [29], thereby enhancing the translational value of experimental studies. Given the crucial role of endothelial cells in SIRS, these cells are one of the primary targets for endotoxin itself and released cytokines, often leading to barrier dysfunction. Based on evidence from experimental studies mainly performed in rodents, LPS may directly or indirectly affect both paracellular and transcellular transport mechanisms at the BBB, including active transport into or out of the brain [30–33]. The impact of LPS-induced meningitis on brain drug disposition was previously studied in pigs [34]. However, to our knowledge, no studies have investigated the impact of systemically administered LPS on brain drug disposition in pigs. Examining CNS drug disposition and systemic pharmacokinetics (PK) in LPS-treated pigs using oxycodone as a model drug provides valuable translational insights, bridging the gap between rodent models and patients, as inflammatory responses vary across species [35].

The gold-standard method to investigate CNS drug disposition is cerebral microdialysis [36, 37]. The technique involves implanting probes at the sites of interest, such as blood, brain tissue, and lateral ventricle (LV). Unlike other methods, it allows for the continuous measurement of unbound concentrations at these sites with high temporal resolution, providing a detailed understanding of drug PK. We therefore used microdialysis in blood, brain interstitial fluid (ISF), and ventricular cerebrospinal fluid (CSF) to characterize the extent of oxycodone transport across the BBB and BCSFB in healthy and endotoxemic pigs. Active net uptake across the barriers is indicated when the ratio between brain/ventricular CSF and blood concentrations (K_p,uu_) is larger than unity [38, 39].

Lumbar CSF sampling is the primary alternative for the characterization of drug CNS exposure. Therefore, to replicate the clinical situation, we longitudinally and simultaneously sampled lumbar CSF, along with brain ISF and ventricular CSF in pigs. This is important, as the reliability of CSF concentrations as a proxy for brain exposure is uncertain and depends on drug properties [40–44].

The overall objective of this study was to assess oxycodone CNS disposition in pigs, to understand inter-species translation of the H^+^/OC antiporter-mediated CNS drug delivery in health and pathology, to facilitate the extrapolation to humans for improved CNS drug treatment in patients. More specifically, the study aimed to: (1) assess the extent of oxycodone delivery across the BBB and BCSFB, (2) investigate the suitability of using lumbar CSF as a surrogate for oxycodone brain ISF concentration, and (3) evaluate the influence of LPS-induced inflammation on oxycodone CNS disposition. Microdialysis plays a pivotal role in achieving these objectives by offering precise, continuous, and direct sampling of unbound drug concentrations from multiple sites, thereby increasing spatiotemporal resolution and significantly advancing our understanding of CNS drug disposition.

## Materials and methods

An extended Materials and Methods section is presented in Supplementary Materials.

### Chemicals

Oxycodone hydrochloride branded as OxyNorm® (Mundipharma, Cambridge, England) and Oxycodone Hameln (Hameln Pharma, Stockholm, Sweden), and saline (0.9 mg/mL NaCl, B. Braun Medical AB, Danderyd, Sweden) were purchased from Distansapoteket Stockholm (Apoteket AB, Stockholm, Sweden). Oxycodone hydrochloride, HPLC grade reference standard (Eur. Qual D, APL, Kungens Kurva, Sweden) was purchased from Distansapoteket (Falun, Sweden). LPS from *Escherichia coli* (O111:B4), oxycodone-D3 and oxycodone-D6 (Cerriliant), ascorbic acid, calcium dichloride, magnesium chloride, potassium chloride, potassium dihydrogen phosphate and sodium chloride were purchased from Sigma Aldrich (Stockholm, Sweden). Artificial brain ISF, CNS Ringer solution (145 mM NaCl, 0.6 mM KCl, 1.2 mM CaCl2, 1 mM MgCl2, 0.2 mM ascorbic acid, KH2PO4 and K2HPO4; pH 7.4) filtered by a 0.45 μm filter (Acrodisc® syringe filter 0.45 μm GHP membrane; Pall Corporation, Port Washington, NY, USA), was prepared in-house. Phosphate buffered saline (PBS: 28 mM Na_2_HPO_4_, 5.6 mM NaH_2_HPO_4_ × 2H_2_O, 95 mM NaCl, in MilliQ water, pH 7.4) filtered by a 0.45 μm filter (Acrodisc® syringe filter 0.45 μm GHP membrane; Pall Corporation, Port Washington, NY, USA), was also prepared in-house.

### Animals

Drug-naïve Swedish landrace pigs of both sexes with body weights of 29.8 ± 1.6 kg, and estimated ages of 10 to 12 weeks, were used in the microdialysis study (n_female_ = 4, n_male_ = 3). Blank plasma from another four drug-naïve pigs of both sexes with body weights of 26.9 ± 0.6 kg was used for determination of the free fraction in plasma (f_u,plasma_) in healthy pigs (n_female_ = 2, n_male_ = 2). The brain from one drug-naïve male pig was used for the determination of a free fraction in brain (f_u,brain_) in a healthy animal.

The study was approved by the Animal Ethics Committee of Uppsala, Sweden (Ethical approval Dnr. 5.8.18-12768/2021), conducted according to regulations of the Swedish Animal Welfare Agency, and in compliance with the European Communities Council Directive of 22 September 2010 (2010/63/EU). The pigs were monitored as intensive care patients by trained personnel throughout the experiment. The study employed the ARRIVE 2.0 guidelines to ensure transparent and comprehensive reporting [45]. The study was not randomized or blinded.

There was no prior information on the neuro-PK of oxycodone in pigs, hence, a pilot study was performed (n = 1), and obtained parameters were used for calculation of the anticipated effect size. Minimally required per-group sample size for a two-tailed t-test study was 6, given the probability level (α = 0.05), the anticipated effect size (Cohen’s d = 1.5), and the desired statistical power level (0.8).

### In vivo study

**Fig. 1:**
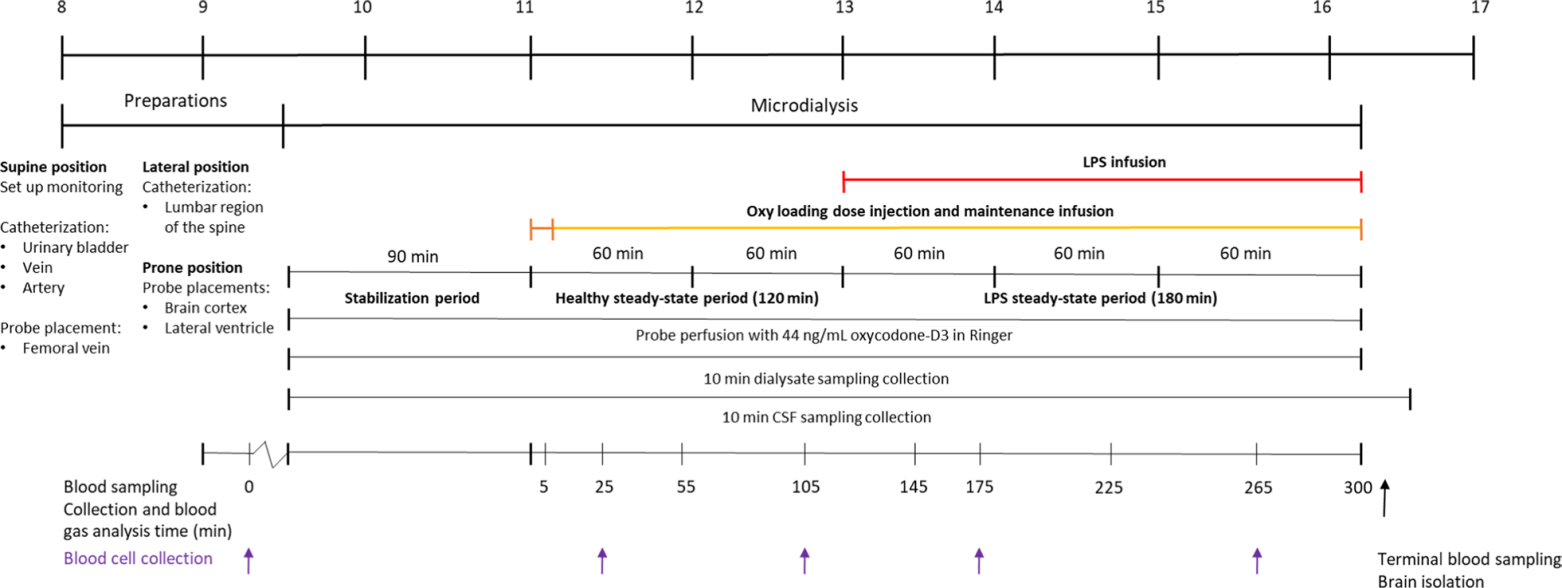
An overview of the experimental design. The pigs were first prepared, including monitoring set-up, catheterizations and probe placements. The experiment was initiated by a 90-min stabilization period, i.e., initiation of probe perfusion. The pigs were administered oxycodone (i.v. loading dose of 0.16 mg/kg oxycodone over two minutes and an i.v. maintenance dose of 0.05 mg/kg/h oxycodone during five hours) followed by a concomitant LPS infusion (i.v. infusion of 4 µg/kg/h LPS from *Escherichia coli*, O111:B4) initiated at 120 min. The probes were perfused with Ringer solution containing oxycodone-D3 at 1 μL/min throughout the experiment. CSF was withdrawn at a rate of 4 µL/min. Dialysate- and CSF samples were collected in 10-min intervals, and blood was sampled at the specified time points. BCs were sampled at specified time points indicated with arrows. The brain was isolated terminally. The top bar indicates the time of the day (24-h clock)

An overview of the study design is presented in Fig. 1. The experiment lasted for eight hours and was divided into four different stages; briefly, (i) preparation of the pigs and implantation of microdialysis probes and intrathecal catheter, (ii) a 90-min stabilization period for conditioning of probes and in vivo microdialysis recovery, (iii) intravenous (i.v.) oxycodone infusion between 0 and 300 min, with 0 to 120 min as a healthy control period, (iv) i.v. LPS administration between 120 and 300 min.

#### Preparation of the pigs

On the day of the experiment, the pigs were transported from the breeder to Uppsala University. Detailed information on the preparation of pigs in presented in Supplementary Materials, Extended Materials and Methods section. A balanced electrolyte solution (Ringer-Acetate Baxter Viaflo) was administered as an i.v. infusion of 10 mL/kg/h for maintenance of healthy hydration throughout the experiment. Noradrenaline 20 µg/mL was administered if needed, starting at 5 mL/h, to maintain a mean arterial pressure (MAP) above 60 mmHg. A blood gas analyzer (ABL 800 flex, Radiometer) was used to monitor acid–base status, electrolytes, hematocrit (Hct), levels of hemoglobin, and glucose.

A small cervical artery was catheterized for blood sampling and blood pressure measurements (Careflow™, Merit Medical Systems Inc., USA). The external jugular vein was catheterized for infusions and blood pressure measurements (multi-lumen central venous catheterization set, blue FlexTip® catheter, CS-15703, Arrow International Inc., Teleflex Inc., USA). An epidural catheter was placed intrathecally in the lumbar region of the spine for the sampling of lumbar CSF (Portex® Epidural catheter, nylon, 3 lateral eyes, 16G, Smiths Medical ASD, Inc. Keene, NH 03431, USA, n = 6) and connected to a pull-pump (REGLO ICC Digital Peristaltic Stand-Alone Pump; 2-Channel, 12 Roller). Microdialysis probes, 10 mm custom-made CMA 20 Elite probe, 60–120 mm shaft, molecular cut off 20 kDa (CMA Microdialysis AB, Kista, Sweden), were implanted in the femoral vein (n = 7), frontal cortical region of the brain (from now on referred to as brain, n = 7) and LV (n = 4) to obtain unbound concentrations in blood, brain ISF and LV CSF. Before placement, the probes were prepared according to the manufacturer’s instructions (CMA Microdialysis AB, Sweden), i.e. placed in Eppendorf tubes containing CNS Ringer solution, and perfused with CNS Ringer solution for a minimum of ten minutes. The probes placed in the CNS were implanted through burr holes over the convexity, fixed using bolt/-s and/or a peripheral venous catheter, and bone wax if needed (COVIDIEN™, BW25G). Sonosite M-Turbo ultrasound (Washington, USA) was used to support the correct placement of the LV probe. The probe positions were visually confirmed terminally. When the preparation was finalized, the pigs were placed in a prone body position.

#### Stabilization period

After the pigs were prepared and before oxycodone was administered, a stabilization period of 90 min was initiated for conditioning of the probes and to allow for recovery of the tissues. At the start of the stabilization period, perfusion of the microdialysis probes with CNS Ringer solution was initiated, and maintained throughout the experiment, using a CMA 400 Syringe Pump (CMA Microdialysis AB, Kista, Sweden). The Ringer solution was spiked with 44 ng/mL oxycodone-D3 to continuously measure the recovery across the probe membrane in vivo, i.e., using the retrodialysis by calibrator approach [46]. To confirm that the relatively short 90-min stabilization period was enough for brain tissue recovery, a microdialysis study was performed according to previously published protocol [10] in one healthy rat. The aim was to investigate the impact of a shorter than 24-h recovery period on the probe recoveries and the extent of BBB uptake. The results showed a minimal impact of a short 90-min recovery period on the assessment of the extent of oxycodone transport across the BBB.

#### Oxycodone- and lipopolysaccharide administrations

Oxycodone steady-state was initiated by an i.v. loading dose of 0.16 mg/kg over two minutes and maintained by an i.v. infusion of 0.05 mg/kg/h throughout the 5-h experiment (Braun volume pump, B. Braun Medical AB, Danderyd, Sweden). The oxycodone doses were based on clinically relevant target steady-state concentration of 60 ng/mL [47]. To estimate pig PK parameters, allometric scaling was applied [48, 49], where human PK were obtained from the literature [47] according to:

1 $${CL}_{human}={CL}_{pig}\times ({{BW}_{human}/{BW}_{pig})}^{0.75}$$

The LPS dose was selected based on a prior study in pigs, where LPS from *Escherichia coli* (O111:B4) was administered intravenously at a rate of 4 µg/kg/h [22]. This dosage resulted in low mortality rates and elevated levels of cytokines such as tumor necrosis factor alfa (TNF-α) and interleukin 6 (IL-6), along with significant circulatory, respiratory, and metabolic changes during 6 h after initiation of LPS administration [23]. This LPS dose has previously been used to induce a septic like state in pigs [22, 23, 25, 26].

Initially, a pilot study was conducted to validate the feasibility of the study, including suitable oxycodone and LPS doses. As the oxycodone concentrations in the blood and CNS compartments were very stable even after initiation of the LPS infusion, the healthy period of 150 min was shortened to 120 min and considered enough as a control period, and by that, the LPS period was extended by 30 min for the following pigs (ID 2–7).

After the healthy period of 120 (n = 6) or 150 min (n = 1), the LPS challenge was initiated as an i.v. infusion of 4 µg/kg/h LPS from *Escherichia coli* (O111:B4, Sigma) throughout the experiment for a total of 180 (n = 6) or 150 min (n = 1) using the Braun volume pump.

To characterize the pathological severity, a pSOFA score was used as a numerical system to evaluate organ failure and septic status [29]. pSOFA score is based on evaluation of organ functions of three domains (3D), i.e., respiratory (PaO_2_/FiO_2_ ratio), cardiovascular (MAP) and renal (urine output) functions. In our study, the 3D-pSOFA score was calculated at 4–6 different time points for each animal to evaluate the degree of organ failure and contribute to the evaluation of the LPS-induced inflammation model.

#### Sample collection

Dialysate samples from the perfused probes in blood, brain and LV were collected in pre-weighed polypropylene microvials (AgnTho’s, Lidingö, Sweden) in 10-min intervals throughout the experiment, using CMA 142 fraction collectors (Harvard Apparatus Inc., Holliston, MA). Lumbar CSF from the intrathecal space was collected in pre-weighed polypropylene microvials in 10-min intervals using the peristaltic pull-pump with a withdrawal rate of 4 µL/min, and a fraction collector (CMA 470 Refrigerated Fraction Collector, Harvard Apparatus Inc., Holliston, MA). Dialysate and CSF samples were immediately capped and stored at 6˚C until bioanalysis the following day.

Blood was sampled in Vacutest® tubes before the start of the oxycodone infusion and at 5, 25, 55, 105, 145, 175, 225, and 265 or 285 min, as well as terminally (Sodium heparin 102 I.U., 6 mL, Polyethylene terephthalate, Vacutest Kima, Arzergrande PD, Italy). At each blood sampling time point, blood was also collected in syringes for blood gas analysis (3 mL, Portex, arterial blood sampling syringe with dry Lithium and Heparin for gases and electrolytes, Smiths medical ASD, Minneapolis, USA).

Terminally, the brain was isolated and regions including the frontal, parietal, and occipital cortices were collected. Upon isolation, the brain was visually examined to confirm the placement of the probes and to ensure no hemorrhages around the probes.

#### In vivo recovery calculation

Retrodialysis by calibrator was performed in vivo throughout the experiment to continuously monitor the recovery of each individual probe over time [46]. The calibrator used was oxycodone-D3, thereby, allowing for the assumption of equivalent recovery characteristics. The recovery was calculated as follows.

2 $$Recovery={(C}_{in}-{C}_{out})/{C}_{in}$$

where C_in_ is the concentration of oxycodone-D3 in the perfusion solution of each probe, sampled from the perfusion syringes prior to and after the experiment procedure, and C_out_ is the concentration of oxycodone-D3 in each dialysate sample.

Unbound oxycodone concentrations (C_u_) in blood, brain ISF and LV CSF obtained by microdialysis were estimated using the probe dialysate sample concentration (C_dialysate_) and the probe recovery, as:

3 $${C}_{u}={C}_{dialysate}/Recovery$$

The mean recoveries in blood, brain and LV were 36.3 ± 11.0% (n = 5), 21.8 ± 6.0% (n = 5) and 33.2 ± 28.1% (n = 3), respectively (Tables S2 and S3). To avoid loss of information and to compensate for recovery changes over time (Fig. S1), a moving average recovery was applied to obtain unbound concentrations over time [46]. Thus, a mean recovery of the calibrator from three subsequent dialysate samples centralized around the sample interval was used [46]. This was applied for all the probes. In pig ID4, the calibrator recovery in all probes was low or negative, yet, with similar oxycodone concentrations in the dialysate samples as the other pigs. For these probes, the moving average recoveries from mean, location-specific, recoveries from the other pigs were applied.

### Oxycodone partition into blood cells

As oxycodone is partitioning into blood cells (BCs) in rats with reported partition ratios between blood and plasma (C_b_/C_p_) of 1.3 ± 0.3 and 1.2 ± 0.1 [5, 10], the C_b_/C_p_ ratio was determined to examine if this phenomenon is preserved in pigs.

### In vitro plasma protein binding and brain tissue binding of oxycodone

Equilibrium dialysis was performed to obtain the fraction of unbound oxycodone in plasma (f_u,plasma_) and brain tissue (f_u,brain_) to evaluate the binding of oxycodone to plasma proteins and to brain tissue in pigs. The technique was performed as previously described [10, 50–52].

### Bioanalysis

Oxycodone and oxycodone-D3 were quantified in the samples using an Acquity ultra-performance liquid chromatography coupled with Xevo TQ-S Micro triple quadrupole mass spectrometer (UPLC-MS/MS, Waters Corporation, Milford, MA, USA) as previously described [10].

### Data analysis of PK parameters

The mean unbound concentration at steady-state (C_u,ss_) and the mean total concentration at steady-state (C_tot,ss_) were calculated in the healthy and LPS-treated periods using samples collected from 55 to 120 or 150 min, and from 120 or 150 to 300 min, respectively, after the start of oxycodone infusion, to ensure that the mean was based on samples collected when plasma steady-state was reached.

As oxycodone is a basic organic cation, it is almost fully ionized at physiological pH. Using the Henderson-Hasselbalch equation [53, 54], and the reported pK_a_ of 9.1 [55], the fraction of ionized oxycodone in the blood was estimated.

Total oxycodone concentrations in blood (C_tot,blood_) were estimated using the total concentration in plasma (C_tot,plasma_) and individual C_b_/C_p_ (Eq. S3) values in the healthy and LPS-treated periods.

4 $${C}_{tot,blood}={C}_{tot,plasma}\times {C}_{b}/{C}_{p}$$

The fraction of unbound oxycodone in blood (f_u,blood_) was estimated as:

5 $${f}_{u,blood}=\frac{{f}_{u,plasma}}{{C}_{b}/{C}_{p}}$$

To evaluate the extent of drug distribution to the CNS, the unbound partition coefficients in brain (K_p,uu,brain_) and lateral ventricle (K_p,uu,LV_) were calculated according to the following equation.

6 $${K}_{p,uu}={C}_{u,CNS,ss}/{C}_{u,blood,ss}$$

where C_u,CNS,ss_ is the mean unbound concentration in the CNS compartment, including brain ISF or LV CSF, at steady-state, and C_u,blood,ss_ is the mean unbound concentration in blood at steady-state. As lumbar CSF was directly sampled according to clinical routine, the K_p,uu_ was calculated using the mean concentration in lumbar CSF (C_u,lumbarCSF,ss_) and total concentration in plasma at steady-state (C_tot,plasma,ss_) corrected for plasma protein binding (f_u,plasma_) as:

7 $${K}_{p,uu,lumbarCSF}={C}_{lumbarCSF,ss}/{(C}_{tot,plasma,ss}{\times f}_{u,plasma})$$

For comparison, the relative extent of drug delivery between two CNS sites was calculated as the ratio of the K_p,uu_ values within each pig, as:

8 $$Extent_{relative}={K}_{p,uu,CNS1}/{K}_{p,uu,CNS2}$$

The total partition coefficient in brain (K_p,brain_) was calculated as a brain-to-plasma concentration ratio using the total concentrations at steady-state. To evaluate the intra-brain distribution in vivo, the apparent unbound volume of distribution (V_u,brain_) was estimated as a ratio between the total amount of drug in brain tissue and unbound concentration in brain ISF. Intra-brain distribution was evaluated by comparison of V_u,brain_ values with physiological volumes in the brain, where a higher V_u,brain_ value than 1 mL/g brain indicates a more extensive brain tissue binding, and/or distribution to cells and subcellular organelles as previously described [56, 57]. V_u,brain_ values were inversed to get an indication of the in vivo f_u,brain_, i.e., f_u,brain_ $\approx$ 1/ V_u,brain_ [55].

To evaluate the systemic PK parameters of oxycodone in pigs, CL was estimated using total plasma concentration–time profiles.

9 $$CL={R}_{0}/{C}_{tot,plasma,ss}$$

where R_0_ is the oxycodone infusion rate and C_tot,plasma,ss_ is calculated at 55–120 min (healthy period) and 120–300 min (LPS period), respectively.

### Statistical analysis

GraphPad Prism version 9.0.0 for Windows (GraphPad Software, San Diego, California, USA) was used for performance of statistical analysis. The normal Gaussian distribution of the data was confirmed using Shapiro–Wilk’s and/or D’Agostino & Pearson normality tests. Paired or unpaired two-tailed t-tests were used for comparisons of the parameters between the healthy and the LPS period, and between methods. Repeated measures two-way ANOVA followed by Šídák’s multiple comparison test were used for comparisons of probe recovery between locations during healthy and LPS periods, and between C_u,ss,LV_ and C_u,ss,lumbarCSF_ during healthy and LPS periods. Mixed-effects analysis with the Geisser-Greenhouse correction followed by Tukey’s multiple comparisons test was used to compare C_tot,brain_ between brain regions, and K_p,uu_ between each probe location in the healthy and the LPS period, respectively. p-values below 0.05 were used to indicate significance. Data are presented as mean ± standard deviation (SD).

## Results

### Physiological status of the pigs during the experiment

**Fig. 2:**
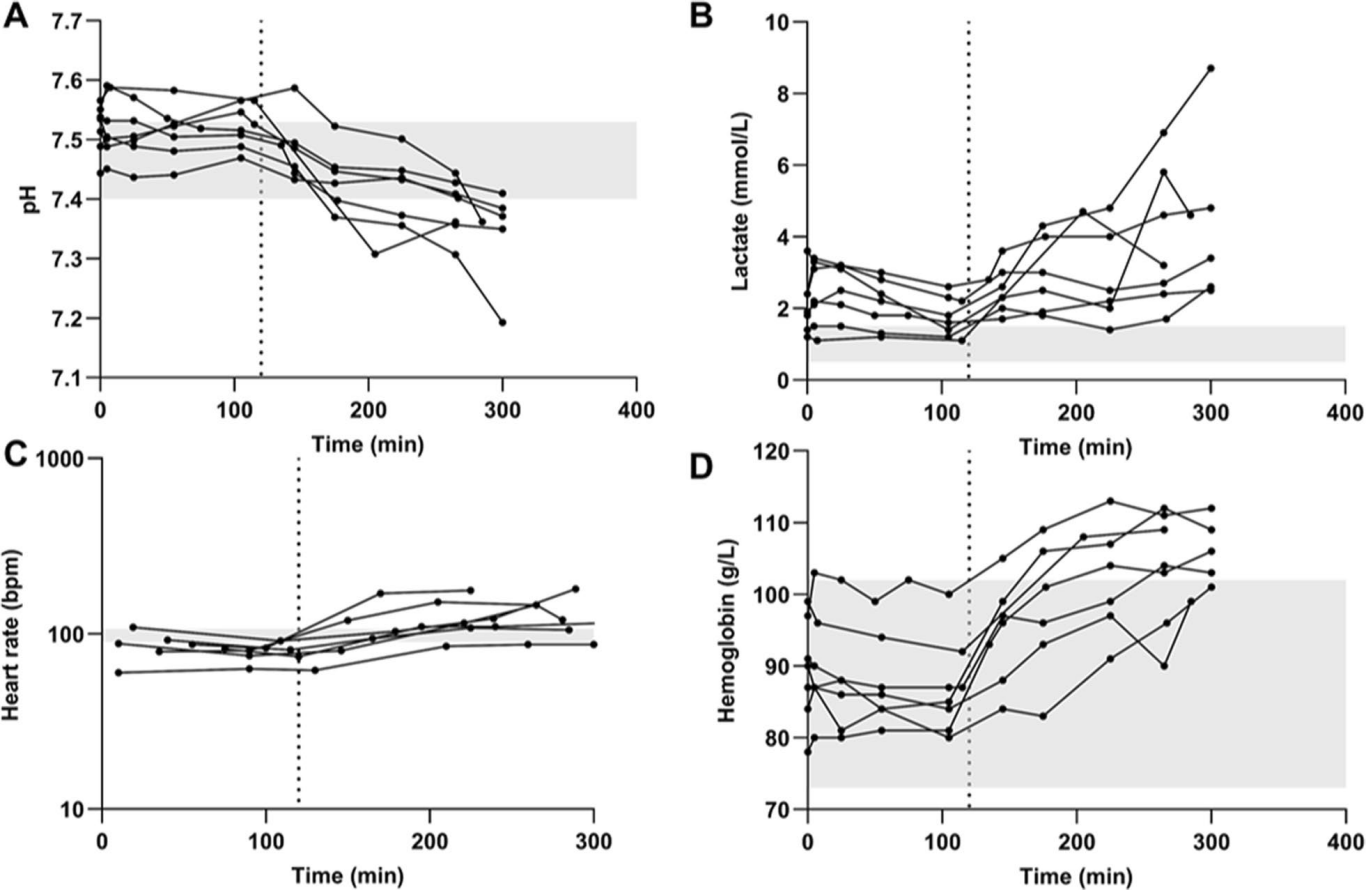
Individual health parameters over time, i.e., **A**. blood pH, **B**. lactate levels in blood, **C**. heart rate, **D**. hemoglobin levels in blood. The shaded areas are normal physiological ranges previously reported in 20–25 kg awake healthy Yorkshire/Duroc cross swine of both sexes [58]. The dotted line at 120 min indicates the initiation of LPS challenge

The pigs were healthy before anesthesia and stable during the preparation and the following stabilization and experimental periods. The administration of LPS effectively induced SIRS in almost all pigs, evidenced by physiological derangements (Fig. 2, Fig. S2). This included decreased blood pH and base excess suggesting combined respiratory and metabolic acidosis, increased plasma lactate levels indicating lactic acidosis, and slightly increased heart rate and hemoglobin levels (Fig. 2, Fig. S2), all consistent with prior observations [22].

Respiration and circulation were maintained within the prespecified limits [58]. No pronounced hypoxia was observed, as a result of the increased oxygen supply on hypoxic indications, and the oxygen saturation was above the recommended lower limit of 92% [59]. The individual mean pSOFA score during the LPS challenge ranged between 3 and 5, confirming a successful induction of a sepsis-mimicking state (Table S1). According to definitions, pig scores between 1 and 3 were categorized as sepsis, and between 5 and 9 as septic shock [29]. One of the pig’s pSOFA scores did not suggest a sepsis-like state (ID 4), with a score of 0 throughout the experiment. Three of the pigs (ID 3, 6 and 7) had scores of 3–4 even from the first measurements taken at 75, 40, 35 min, respectively, after start of oxycodone infusion, but the scores also increased to 4–6 after LPS administration (Table S1).

### Oxycodone is actively transported across the BBB in healthy and LPS-treated pigs

**Fig. 3:**
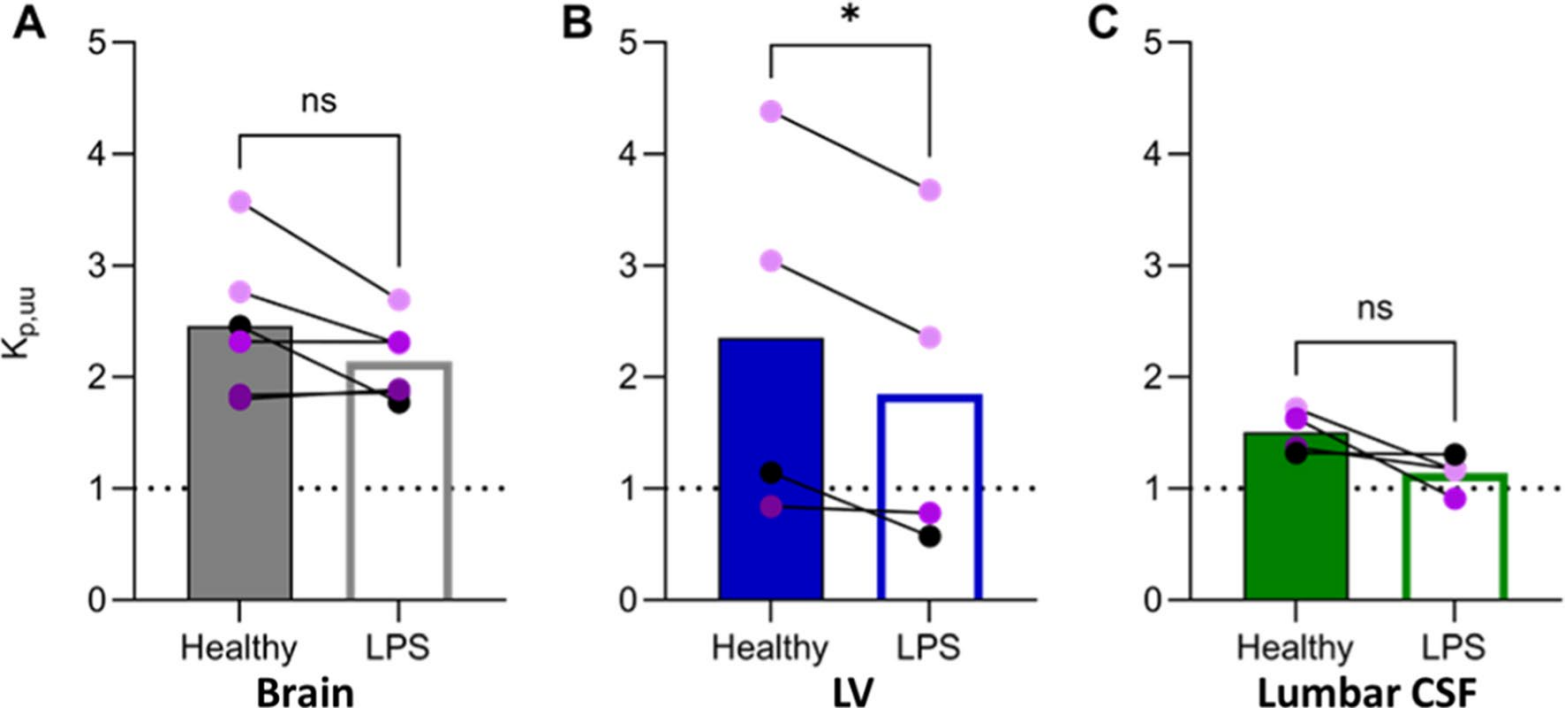
Scatter dot plots of K_p,uu_-values in individual healthy (filled bars) and lipopolysaccharide (LPS)-treated (open bars) states, in A. brain, B. lateral ventricle (LV) and C. lumbar cerebrospinal fluid (CSF). The colors of the individual values are based on the SOFA score, the darker purple the higher the score. SOFA score of 0 (black), 3 (light purple), 4 (intermediate purple), and 5 (dark purple). Statistical test details are presented in Table 1

**Fig. 4:**
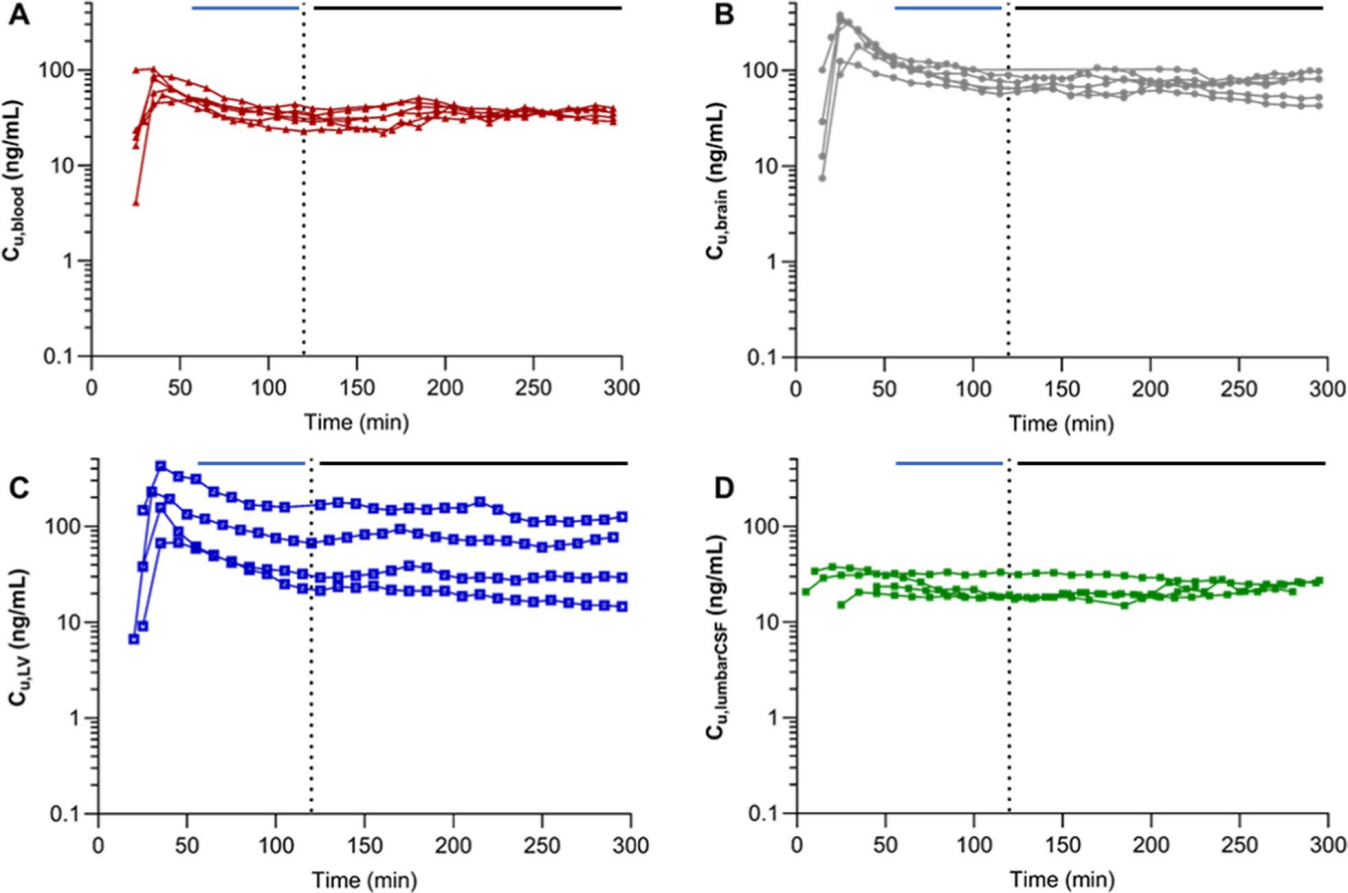
Unbound concentration–time profiles of oxycodone in **A**. blood, **B**. brain, **C**. lateral ventricle (LV) obtained with microdialysis and D. lumbar cerebrospinal fluid (CSF). An i.v. loading dose of 0.16 mg/kg oxycodone was administered over two minutes and an i.v. maintenance dose of 0.05 mg/kg/h oxycodone during five hours. The dotted line at 120 min indicates the initiation of LPS challenge. The solid horizontal lines indicate the periods of the samples included to calculate mean unbound concentrations at steady-state during the healthy (blue, 55–120 min) and the LPS-treated (black, 120–300 min) periods

Active net uptake of oxycodone was observed at the BBB in the anaesthetized pigs of both sexes during the healthy state, with a mean K_p,uu,brain_ value of 2.5 (Table 1, Fig. 3). The unbound systemic and brain ISF concentrations were stable over time (Fig. 4). This finding suggests that the H^+^/OC antiporter system previously characterized in rodents, may also be present and functionally active at the BBB in pigs, supporting the potential conservation of this transport mechanism across species.

**Table 1:** Site-specific characterization of oxycodone pharmacokinetic parameters

	Healthy	LPS	p-value
Site-specific concentrations			
C_u,blood,ss_ (n = 6)	37.2 ± 6.3	34.9 ± 3.6	> 0.05
C_u,brain,ss_ (n = 6)	89.3 ± 18.3	73.4 ± 13.9	**0.006**
C_u,LV,ss_ (n = 4)	86.4 ± 70.0	67.1 ± 56.5	> 0.05
C_u,lumbarCSF,ss_ (n = 4)	23.3 ± 6.2	22.1 ± 4.4	> 0.05
Site-specific extent of transport			
K_p,uu,brain_ (n = 6)	2.5 ± 0.7	2.1 ± 0.4	> 0.05
K_p,uu,LV_ (n = 4)	2.4 ± 1.7	1.8 ± 1.5	**0.04**
K_p,uu,lumbar CSF_ (n = 4)	1.5 ± 0.2	1.1 ± 0.2	> 0.05

Mean unbound oxycodone concentrations in blood, brain, lateral ventricle (LV) and lumbar cerebrospinal fluid (CSF), at steady-state during the healthy and lipopolysaccharide (LPS)-treated conditions, reported in ng/mL. K_p,uu_ in brain, LV and lumbar CSF during the healthy and LPS-treated conditions

Mean ± SD. C_tot,blood_ is calculated using Eq. 4., and C_u_ in blood, brain and LV are calculated using Eq. 3. K_p,uu_ estimated using Eqs. 6–7. p-values present t-test results on statistical comparison of C_u,ss_ and K_p,uu_ between the healthy and LPS periods. Further statistical comparisons are presented in Tables S4

**Fig. 5:**
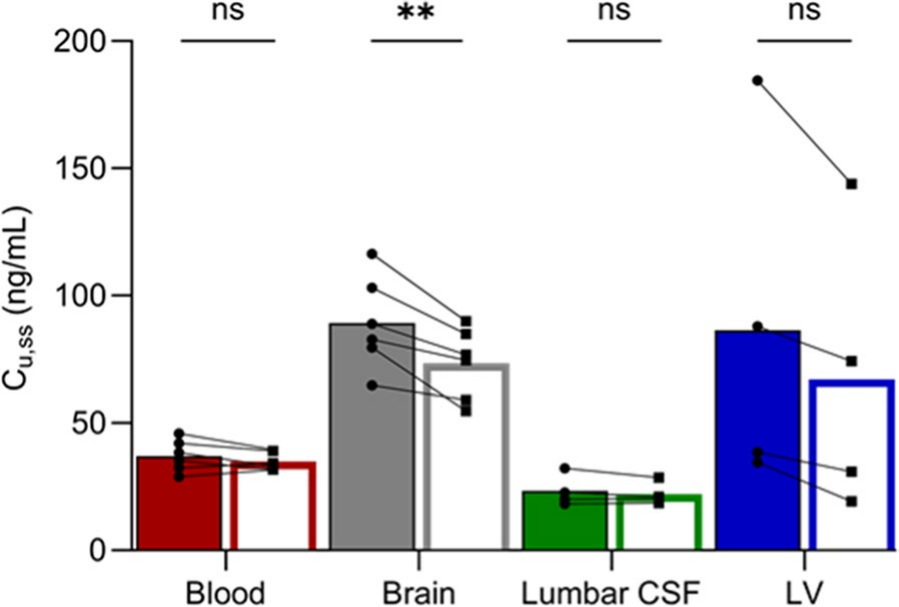
Unbound concentrations at steady-state (C_u,ss_) in blood, brain, lumbar and ventricular (LV) cerebrospinal fluid (CSF) during the healthy state (filled bars) and lipopolysaccharide challenge (open bars). The bars indicate the mean values. Paired t-tests were used to compare C_u,ss_ during the healthy and LPS period in each compartment, **p < 0.01, ns p > 0.05

During LPS challenge, unbound plasma concentrations remained consistent with those observed during the healthy state (p = 0.20), while the concentrations in the brain (C_u,brain,ss_) were significantly lower (p = 0.006, Fig. 4, 5, Table 1). Yet, the mean oxycodone uptake at the BBB with a K_p,uu,brain_ of 2.1 did not reach a significant decrease during the LPS challenge (p = 0.12, Fig. 3, Table 1, Fig. S3).

Oxycodone, as a basic organic cation, is almost fully ionized at physiological pH. Therefore, changes in blood pH induced by LPS administration (Fig. 2A) may affect its ionization state and potentially impact the BBB transport. During the LPS-treated condition, the estimated fraction of ionized oxycodone in blood was 98.0 ± 0.2%, which was significantly higher than the 97.4 ± 0.2% observed under healthy conditions (p = 0.006). Despite minimal changes in the ionization state, the absolute blood concentrations of unbound and unionized oxycodone, which can cross biological membranes by passive diffusion route, remain very low in both conditions.

### Variable extent of oxycodone delivery to the cerebrospinal fluid sites

Compared to the extent of BBB transport, there was a substantial variation in the measured extent of uptake of oxycodone across the BCSFB, with K_p,uu,LV_ ranging between 0.8 and 4.4 (Table 1, Fig. 3). Nevertheless, the mean K_p,uu,LV_ of 2.4 indicated active uptake of oxycodone at the BCSFB in healthy pigs.

Monitoring of oxycodone in lumbar CSF revealed minimal inter-individual variability with a mean K_p,uu,lumbarCSF_ of 1.5 in the healthy state (Table 1, Fig. 3). There was a trend indicating that mean lumbar CSF concentrations were lower than those in the LV, yet not significant mainly due to the high variability in the LV data. The same non-significant trend was present when comparing K_p,uu,lumbarCSF_ and K_p,uu,LV_. However, K_p,uu,lumbarCSF_ was significantly lower than K_p,uu,brain_ in both healthy and LPS conditions (Table S2).

The individual relative extent of oxycodone uptake at the BBB compared with that at the BCSFB was variable, but on average, K_p,uu_ was 1.5-fold higher at the BBB compared with that at the BCSFB, with a K_p,uu,brain_-to-K_p,uu,LV_ ratio range of 0.6–2.1 (CV 49.3%, n = 4). However, the difference was not significant (Fig. 3).

The mean unbound oxycodone in the lateral ventricle, C_u,LV,ss_, and the extent of oxycodone uptake at the BCSFB was lower during LPS challenge (76 ± 15% of the healthy control), while there was no significant change in lumbar CSF oxycodone concentrations (p = 0.30, Figs. 3, 4, 5, Table 1). Despite the large inter-individual variability in K_p,uu,LV_, a consistent decrease was observed during the LPS challenge, with K_p,uu,LV_ being 76 ± 18% of the healthy value (p = 0.04). The same trend was observed for K_p,uu,lumbarCSF_, where the ratio during LPS challenge to that in healthy pigs was 77 ± 19% (p = 0.11).

### Systemic pharmacokinetics of oxycodone in pigs

The blood oxycodone concentration–time profiles were stable over time and did not change by LPS challenge (Figs. 4 and 5, Table 1). The oxycodone CL, based on total plasma concentrations, was therefore similar during the healthy and the LPS-treated periods, estimated to be 47 ± 9 and 41 ± 8 mL/min/kg, respectively (p = 0.13). There were no tendencies of sex differences in the systemic PK of oxycodone.

The hematocrit was increased by LPS challenge, with values of 27.5 ± 2.1% and 31.4 ± 2.3% during the healthy and LPS periods, respectively (n = 7, p = 0.0014). However, both of these values were within the normal physiological range [58]. The partition of oxycodone to BCs (C_b_/C_p_) was 1.2 ± 0.09 (N = 7, n = 1–2), without any change during LPS challenge (p = 0.08). The f_u,plasma_ values obtained by equilibrium dialysis during the healthy and LPS-treated periods were 88.5 ± 7.3% and 85.7 ± 6.4% (n = 7, p = 0.44), respectively. Similarly, the f_u,blood_ values, estimated using each individual’s f_u,plasma_ and C_b_/C_p_-values, were 74.9 ± 6.6% (n = 6) during the healthy state, and 69.5 ± 8.9% (n = 7) during the LPS state (p = 0.27).

### Intra-brain distribution

Terminally, brain tissue samples were collected from the frontal, parietal and occipital cortices, showing similar total oxycodone concentrations with a mean value of 112 ± 16 ng/mL. In one of the pigs, spinal cord tissue was also sampled. In this pig, the brain oxycodone concentration in the different cortical regions were 91.5–96.3 ng/mL, while the spinal cord was slightly lower at 80.0 ng/mL. Total steady-state concentrations in blood of 26.1 ± 5.9 ng/mL, resulted in total partition coefficients between brain and blood (K_p,brain_) from the three brain regions of 4.0 ± 1.0 (n = 7). The individual total oxycodone concentration in the frontal cortex sample, and the unbound concentration obtained by the brain probe placed in the frontal cortical area, were used to estimate the V_u,brain_ value of 1.69 ± 0.56 mL/g brain (n = 6). The latter indicated non-restricted intra-brain distribution of oxycodone governed by non-specific brain tissue binding and uptake into the parenchymal cells. The brain tissue binding estimate obtained from the inverted in vivo-estimated V_u,brain_ (1/V_u,brain_), was not significantly different from the in vitro f_u,brain_ obtained by equilibrium dialysis of 48.3 ± 14.9% (n = 7, p = 0.15). In the healthy pig brain not having received LPS, the in vitro f_u,brain_ was estimated to be 67.3 ± 13.0% (n = 1, in triplicates), which was not statistically different to the value obtained from the LPS-treated pigs of 48.3 ± 14.9% (n = 7, in duplicates, p = 0.09).

## Discussion

The primary objective of this study was to advance our understanding of active drug uptake at brain barriers to higher species than rodents, by examining oxycodone brain concentrations in pigs in healthy and LPS-induced sepsis-mimicking conditions. Additionally, we sought to evaluate the efficacy of lumbar CSF exposure readout as a proxy for brain unbound ISF concentrations. To our knowledge, this is the first comprehensive study characterizing oxycodone CNS disposition in pigs. By simultaneously monitoring concentrations in blood, the frontal cortical area, the lateral ventricle using microdialysis, and the lumbar intrathecal space by direct sampling, we achieved exceptional spatiotemporal resolution in mapping oxycodone transport across the different CNS barriers. Our study demonstrates novel evidence of higher unbound oxycodone concentrations in the brain ISF compared to those in the blood, resulting in a K_p,uu,brain_ value of 2.5. This supports our hypothesis of the presence of the H^+^/OC antiporter system at the BBB in pigs. The observed active net uptake across the BBB in pigs suggests a similar mechanism may exist in humans, indicating the potential for targeted CNS drug delivery.

The K_p,uu,brain_ in pigs is however lower compared with that of 3–4.4 previously reported in healthy rats [5, 10]. This is interesting, as species comparisons of the efflux transporter P-glycoprotein (P-gp) showed less activity in higher species than in rodents [60, 61]. That difference was explained by lower protein expression levels of efflux transporters at the human BBB, including P-gp and breast cancer resistance protein, compared to those in rats [62]. Thus, a similar phenomenon may be present for the H^+^/OC antiporter system proteins. To our knowledge, there is only one study addressing the inter-species BBB transport mediated by the H^+^/OC antiporter system for another substrate, diphenhydramine [63]. Shaffer et al. reported active net brain uptake of diphenhydramine with a similar extent of transport across the BBB in rats, dogs, and non-human primates with mean unbound brain-to-plasma concentration ratios of 3.9, 4.9 and 5, respectively [63]. Another recent study revealed active uptake of diphenhydramine in Göttingen minipigs with K_p,uu,brain_ of 2.2 [64]. In line with our findings, this strengthens the presence of species-independent active BBB uptake mediated by the H^+^/OC antiporter system.

In considering the value of CSF sampling, two primary questions emerge (i) the presence of the H^+^/OC antiporter system at the BCSFB and (ii) the relevance of lumbar CSF readouts as a proxy for the estimation of the unbound brain ISF concentrations. The mean K_p,uu,LV_ of 2.4 indicate that active uptake of oxycodone also exists at the pig BCSFB, with a similar capacity as at the BBB. Although the mean K_p,uu,LV_ values indicate active uptake, we observed extensive inter-individual variability. The ultrasound-guided probe positioning in LV was confirmed by CSF-like fluid observed at the probe insertion site. However, the placement of the LV probe was technically challenging even with the support of ultrasound, possibly contributing to the observed variability. In comparison, the extent of BCSFB transport of oxycodone in pigs was similar to the one reported in rats with K_p,uu,LV_ of 3.4 [10].

Interestingly, there was a trend of lower unbound concentrations in lumbar CSF compared with those in the LV (K_p,uu,lumbarCSF_ 1.5), indicating that the site of sampling plays a crucial role. However, in spite of the difference between the mean K_p,uu_ at the two sites being around twofold, the difference is not statistically significant due to the high variability in LV concentrations. In our study, the withdrawal rate of CSF was one-tenth of previously estimated CSF production rate in pigs of 67 µL/min [65, 66]. Therefore, the impact of withdrawal on the results can be considered to be minimal. Since lumbar sampling is used in humans, this study along with previous reports, suggests that CSF concentrations should not be used as a proxy for unbound concentrations in brain ISF without verification of their relationship, in particular, if a drug is a substrate of a drug transporter [40, 42, 67].

In addition, in the study reporting oxycodone exposure in lumbar CSF and plasma after i.v. administration of oxycodone to women undergoing elective gynaecological surgery, we could see a similar trend to that observed in pigs [68]. That is, the ratio between the reported mean AUCs in lumbar CSF (185 h ng mL ^−1^) and total plasma (157 h ng mL ^−1^) was 1.2 which was similar to the 1.3 ratio between C_u,lumbarCSF,ss_ (23.3 ng mL ^−1^) and C_tot,plasma,ss_ (18.3 ng mL ^−1^) in the present study. Unfortunately, plasma protein binding was not reported for female patients by Kokki et al., which makes it difficult to estimate absolute values of K_p,uu,lumbarCSF_ in humans. However, by applying reported f_u,plasma_ in humans of 38–45% [69], we could predict that K_p,uu,lumbarCSF_ in these patients is higher than unity, indicating a similar direction as that observed in pigs. Hence, we suggest that lumbar CSF is a reasonable translational readout for oxycodone exposure between pigs and patients.

Remarkably, despite extensive changes in the physiological status of pigs reflected in pSOFA scores, nearly unchanged blood concentrations of oxycodone were documented within three hours of i.v. infusion of LPS. However, the unbound brain concentrations decreased by approximately 20% post-LPS administration. The phenomenon of slightly decreased active uptake was also observed at the BCSFB in the LV and lumbar CSF, although only significant at the LV site. If it is true that the extent of oxycodone delivery to the brain is decreased in endotoxemia conditions also in humans, the opioid doses in critically ill patients may need to be somewhat adjusted to avoid under-dosing.

This seemingly unexpected finding of a minor impact of LPS on oxycodone active uptake may have various explanations. It is well known that LPS affects the innate and adaptive immune responses in a dose- and time-dependent manner [21, 22]. Our intention to characterize oxycodone CNS disposition in healthy and LPS-induced inflammation states within the same animal restricted our observational time frames. Yet, within the same animal, we were able to monitor and characterize the early response phase to LPS. It was shown that after initiation of i.v. infusion of 4 µg/kg/h of LPS the blood concentrations of TNF-α reached its maximum already during the first hour and then returned to baseline at three hours, while IL-6 levels were gradually increasing and reached maximum at three hours [22]. As shown in mice, LPS does not virtually enter the brain via the BBB [70], yet it may interact directly with Toll-like receptors expressed in brain microvasculature endothelial cells [71] or indirectly trigger various inflammatory cascades, including cyclooxygenase signaling which may contribute to alterations in BBB function [72, 73]. The direct impact of LPS on the BBB and in particular its impact on the transport of organic cations, is not fully understood and is mainly investigated in in vitro studies. Indirectly via released cytokines that can cross the BBB via saturable selective and specific mechanisms [74], LPS may compromise the BBB function. The latter has been shown by multiple studies in mice and rats, though with many controversies [75, 76]. Comparison of response induced by LPS in the pig brain to data in a similar mouse model demonstrated some overlapping changes and gene sets but also numerous striking differences that may in part explain only slight changes in the CNS disposition of oxycodone post-LPS administration [77].

The net BBB transport of oxycodone results from a complex interplay between paracellular, passive, and active transport mechanisms, each of which may be differentially affected by pathological conditions. Under normal physiological conditions, paracellular transport across the healthy BBB is highly restricted. However, in pathological states like endotoxemia, this route may become compromised. For instance, studies in endotoxemic pigs suggest that paracellular permeability is altered, as indicated by the intracellular astrocytic protein S100B, a biomarker of BBB integrity. These studies have shown transient changes in S100B levels in the blood following LPS infusion at 1 µg/kg/h and 10 µg/kg/h doses, suggesting a temporary mild disruption of the BBB [24, 78]. Despite this potential compromise in BBB integrity, our results indicate that active transport remains a significant contributor to oxycodone’s overall net flux across the BBB as K_p,uu_ is higher than unity. Active transport at the BBB involves both efflux and influx processes. In the case of active efflux, P-gp has been implicated as a potential transporter of oxycodone. Although P-gp is expressed in pigs at levels similar to humans, albeit lower than in rodents [79], the precise role of P-gp in oxycodone transport remains unclear. Some studies propose that oxycodone may be a substrate for P-gp [80–82], while others report no significant impact of P-gp inhibition on its pharmacokinetics or pharmacodynamics [83]. Additionally, there is evidence suggesting that oxycodone may moderately induce P-gp expression [80]. The complexity of this interaction is further heightened by the modulatory effects of endotoxemia on P-gp function. For example, in porcine brain endothelial cells, P-gp activity has been shown to increase in response to elevated levels of interleukin 1β [84], a cytokine whose plasma concentrations rise following LPS administration. However, whether this P-gp modulation occurs in vivo during endotoxemia in pigs remains unconfirmed. If such upregulation does occur, it could partially explain the observed decrease in oxycodone’s net flux across the BBB.

Moreover, the slight reduction in CNS delivery of oxycodone observed in our study may also be attributable to a decrease in active uptake mechanisms. To date, the only identified active uptake pathway for oxycodone involves the H^+^/OC antiporter system. Whether the reduction in the K_p,uu_ seen in LPS-challenged pigs is due to a diminished function of this antiporter system or an upregulation of P-gp remains unresolved. Further in vivo studies are required to elucidate these mechanisms. To our knowledge, there are no studies investigating the CNS delivery of organic cations in pigs in endotoxemia conditions, making it difficult to compare and generalize our findings.

To our knowledge, this study is also the first to provide systemic PK of oxycodone in pigs, with an estimated clearance of 47.2 mL/min/kg. The reported CL in rats was 101 mL/min/kg [10] compared to that reported in humans of 11.5 mL/min/kg [47]. Oxycodone is metabolized by CYP2D6 and CYP3A4 [85, 86]. Studies comparing the drug metabolism between pig- or minipig-, and human liver microsomes concluded similar quantitative activity of CYP3A4 but more extensive and rapid CYP2D6 activity in pigs compared to humans [87, 88].

## Conclusions

This study highlights the importance of inter-species translational studies in vivo for the understanding of CNS drug delivery using microdialysis, allowing unbound concentrations to be measured. It implies that the H^+^/OC antiporter system may also be present in pigs, reflected in an active net uptake to the brain. We have extended the understanding of the impact of host defense mechanisms occurring during the early response to endotoxemia on CNS disposition of oxycodone in pigs. This reveals minimal changes in the extent of unbound oxycodone transport across the BBB and BCSFB. We show that CSF may underestimate unbound brain ISF concentrations in pigs and confirm that the use of lumbar CSF as a surrogate brain is not to be recommended without verification of their relationship. However, lumbar CSF could be a reasonable translational exposure readout between pigs and humans. The findings in pigs are crucial for translational interpretation of CNS drug delivery, when extrapolating preclinical results to humans, thus advancing the understanding of CNS drug treatment for patients.

## Supplementary Information

Additional file 1.

## Data Availability

Data are available from the corresponding authors upon reasonable request.
