# Supplementary material for: Active CNS delivery of oxycodone in healthy and endotoxemic pigs
Source: Fluids Barriers CNS. 2024 Oct 23;21:86. doi: 10.1186/s12987-024-00583-z (PMC11515623; doi:10.1186/s12987-024-00583-z)
Supplement: Supplementary file 1 — Additional file 1. [file 12987_2024_583_MOESM1_ESM.docx]

# Supplementary Materials

## Tables (supplementary materials)

**Table S1. pSOFA score over time.** At each time point, the score is the sum of the scores of the three domains: respiratory (PaO_2_/FiO_2_ ratio), cardiovascular (MAP) and renal (urine output) functions. Lipopolysaccharide challenge was initiated at 150 minutes for pig 1 and at 120 minutes for pig 2-7. Before LPS challenge, during the healthy period, the scores were based on data collected at 2-3 time points in each pig. The score values were consistent within each animal during the healthy period and, hence summarized from 0-120-minute period.

| Time (min) | Pig 1, female | Pig 2, male | Pig 3, female | Pig 4, male | Pig 5, female | Pig 6, male | Pig 7, female |
| --- | --- | --- | --- | --- | --- | --- | --- |
| 0-120 | 0 | 0 | 3 | 0 | 0 | 3 | 4 |
| 120 |  |  | 3 |  |  |  |  |
| 130 |  |  |  | 0 |  |  |  |
| 146 | 0 |  |  |  |  |  |  |
| 150 |  |  |  |  |  |  | 6 |
| 165 |  |  |  |  | 3 |  |  |
| 170 |  |  |  |  |  | 3 |  |
| 179 |  | 3 |  |  |  |  |  |
| 195 |  |  | 3 |  |  |  |  |
| 205 |  |  |  |  |  |  | 5 |
| 210 |  |  |  | 0 |  |  |  |
| 221 | 6 |  |  |  |  |  |  |
| 225 |  |  |  |  | 3 | 4 |  |
| 239 |  | 3 |  |  |  |  |  |
| 240 |  |  | 3 |  |  |  |  |
| 260 |  |  |  | 0 |  |  |  |
| 265 |  |  |  |  |  |  | 5 |
| 281 | 4 |  |  |  |  |  |  |
| 285 |  |  |  |  | 4 |  |  |
| 289 |  | 4 |  |  |  |  |  |
| 300 |  |  |  | 0 |  |  |  |
| 305 |  |  | 4 |  |  |  |  |

**Table S2. Statistical test details of K_p,uu_ comparisons.** Comparisons of the unbound partition coefficient (K_p,uu_) between brain and lumbar cerebrospinal fluid (CSF) in the healthy and the lipopolysaccharide (LPS)-treated conditions.

| **Repeated measures two-way ANOVA, followed by Šídák's multiple comparisons test** | | |
| --- | --- | --- |
| **Number of families** | 1 | |
| **Number of comparisons per family** | 2 | |
| **Alpha** | 0.05 | |
| **Šídák's multiple comparisons test** | | |
| **Brain vs Lumbar CSF** | | |
|  | **Healthy** | **LPS** |
| **Mean Diff.** | 0.95 | 0.9997 |
| **95.00% CI of diff.** | 0.2605 to 1.640 | 0.3101 to 1.689 |
| **Below threshold?** | Yes | Yes |
| **Summary** | ** | ** |
| **Adjusted P Value** | 0.0073 | 0.005 |
| **Test details** | | |
| **Mean 1** | 2.458 | 2.138 |
| **Mean 2** | 1.508 | 1.138 |
| **Mean Diff.** | 0.95 | 0.9997 |
| **SE of diff.** | 0.2796 | 0.2796 |
| **N1** | 6 | 6 |
| **N2** | 4 | 4 |
| **t** | 3.398 | 3.576 |
| **DF** | 16 | 16 |

**Table S3. Overview of probe recoveries (%) during the healthy period, LPS-treated period and the full experiment.**

|  | Healthy | LPS | Full experiment |
| --- | --- | --- | --- |
| Blood probe (n=5) | 40.0±18.8 | 34.1±6.0 | 36.3±11.0 |
| Brain probe (n=5) | 22.3±6.3 | 21.6±6.5 | 21.8±6.0 |
| LV probe (n=3) | 33.3±28.7 | 31.9±28.3 | 33.2±28.1 |

Mean±SD. Paired t-tests showed no difference in mean recoveries between the healthy and the LPS periods for blood (p=0.4) and brain (p=0.6), but the mean LV recovery was lower during the LPS period (p=0.04).

## Figure legends (supplementary materials)

**Figure S1. Probe recoveries in blood (red), brain (gray) and lateral ventricle (LV, blue) over time.** Mean recoveries calculated during the whole experiment are indicated as dotted lines on the y-axis. The probe recoveries were generally decreasing over time, yet, only significant in the LV (blood p=0.40, brain p=0.61, LV p=0.04). The largest changes in recovery over time were present in two blood probes with coefficients of variation (CV) > 30 %. The mean CV of all probes were 16.3 %.

**Figure S2. Health parameters over time.** A. partial carbon dioxide (CO_2_) pressure in blood, B) glucose levels in blood, C. base excess in blood, D. mean arterial pressure, E. body temperature, and F. oxygen (O_2_) saturation in blood. Glucose levels were slightly decreasing with time but still within the interval considered normal. None of the pigs had pronounced low blood pressure. The body temperature was within the healthy interval for pigs, except for one pig with lower temperature (ID4), which may be due to incorrect measurements or other technical deviations during the experiment, and was therefore excluded in the graph. The CO_2_ partial pressure was slightly increasing, but did not exceed the interval considered normal (1). The dotted line at 120 minutes indicates the initiation of LPS challenge.

**Figure S3. Unbound concentration ratios over time.** Unbound concentration ratios in brain (gray), lateral ventricle (LV, blue) and lumbar cerebrospinal fluid (CSF, green), to that in blood. Mean K_p,uu_ (based on C_u,ss_ calculated from 60 to 300 minutes, i.e., full experiment) are indicated as dotted lines on the y-axis. The starting time of lipopolysaccharide infusion is indicated as a dotted line at 120 minutes.

## Figures (supplementary materials)


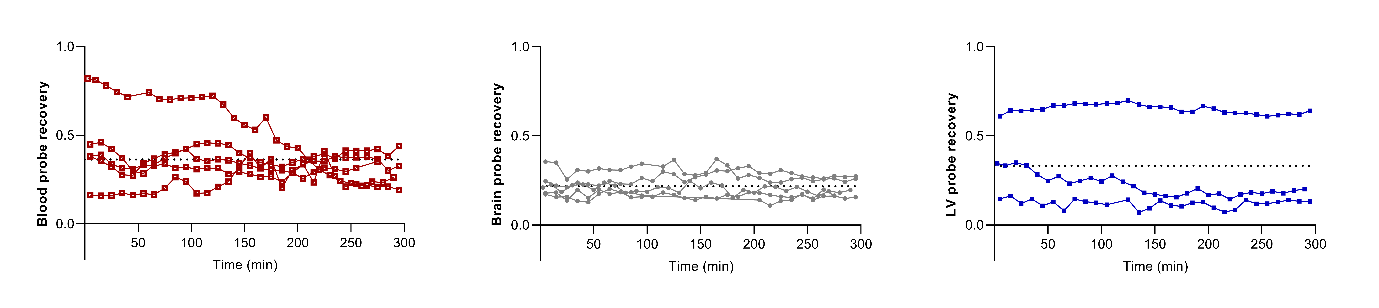


#### Figure S1.


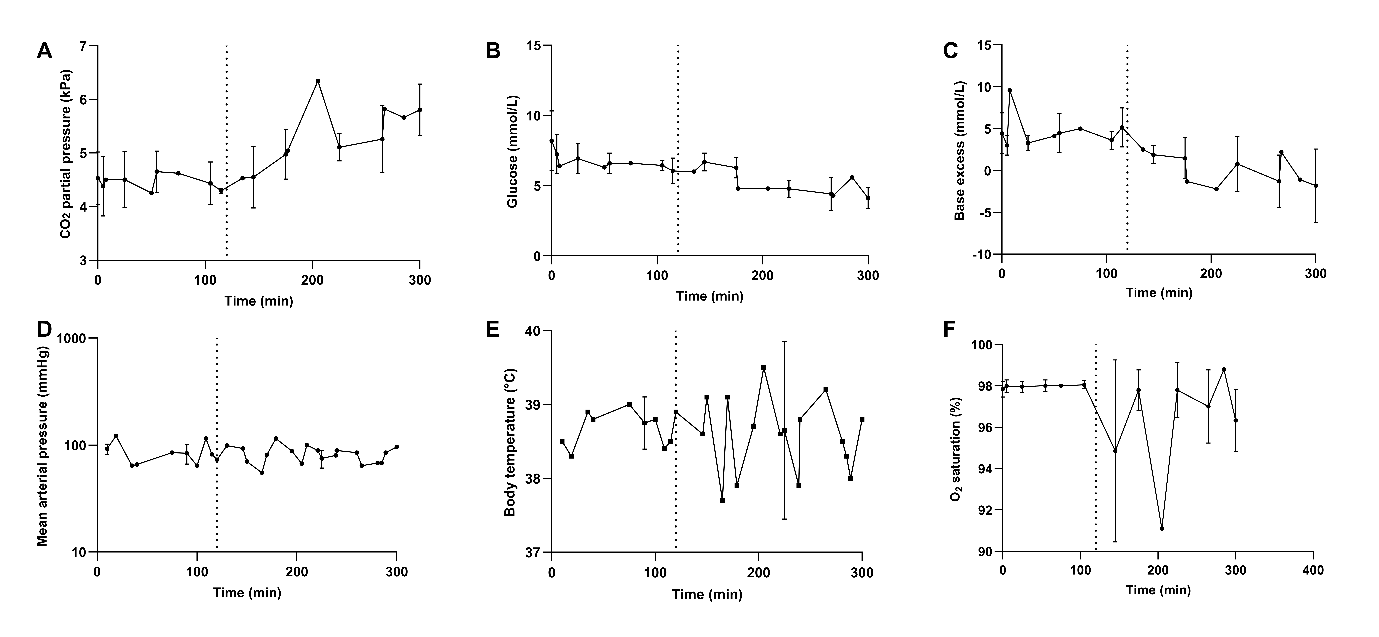


#### Figure S2.


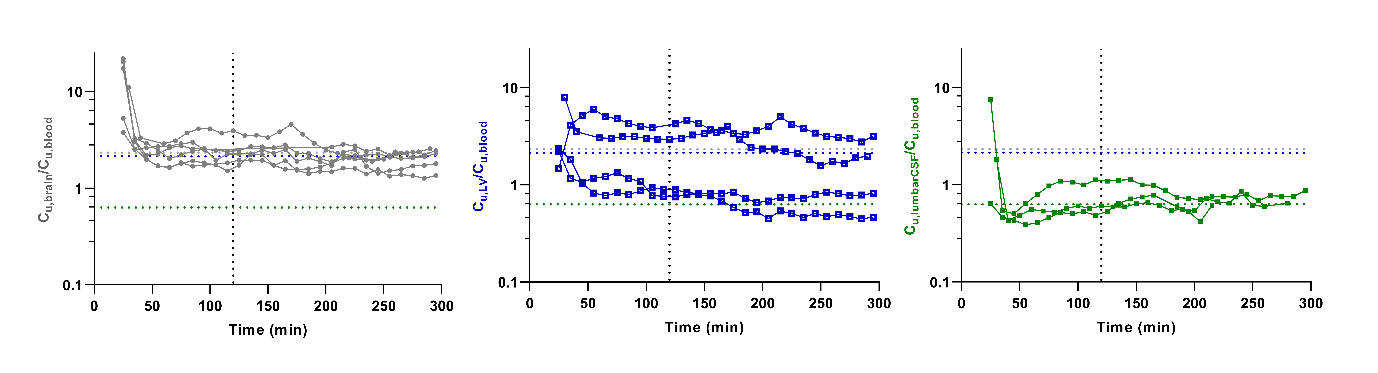


#### Figure S3.

## Supplementary Materials and Methods

### Chemicals

Oxycodone hydrocloride branded as OxyNorm® (Mundipharma, Cambridge, England) and Oxycodone Hameln (Hameln Pharma, Stockholm, Sweden), and saline (0.9 mg/mL NaCl, B. Braun Medical AB, Danderyd, Sweden) were purchased from Distansapoteket Stockholm (Apoteket AB, Stockholm, Sweden). Oxycodone hydrochloride, HPLC grade reference standard (Eur. Qual D, APL, Kungens Kurva, Sweden) was purchased from Distansapoteket (Falun, Sweden). LPS from *Escherichia coli* (O111:B4), oxycodone-D3 and oxycodone-D6 (Cerriliant), ascorbic acid, calcium dichloride, magnesium chloride, potassium chloride, potassium dihydrogen phosphate and sodium chloride were purchased from Sigma Aldrich (Stockholm, Sweden). Acetonitrile (gradient grade for liquid chromatography), dipotassium hydrogen phosphate, formic acid (98–100%), disodium phosphate, disodium phosphate dihydrate, 2-methylbutane (isopentane) were purchased from Merck (Darmstadt, Germany). Milli-Q water was obtained from a Milli-Q Academic system (Millipore, Bedford, MA, USA; Resistance 18.2 Ohm; Millipak®Express 20 Filter, 0.22 μm) from Merck Millipore (Burlington, MA, USA). Ketamine (Ketaminol® vet., 100 mg/mL, Merck Sharp & Dohme (MSD) Animal Health Sweden AB, Sweden), xylazine (Rompun® vet., Elanco Denmark) and tiletamine/zolazepam (Zoletil® 50 mg/mL+50 mg/mL, Virbac, France) were purchased from Swevet, Sjöbo, Sweden. Ringer-acetate (Ringer-Acetate Baxter Viaflo: calcium chloride dihydrate 0.29 g/L, potassium chloride 0.3 g/L, magnesium chloride hexahydrate 0.2 g/L, sodium acetate trihydrate 4.08 g/L, sodium chloride 5.86, Baxter Viaflo, Baxter Medical AB, Kista, Sweden), midazolam (Midazolam Hameln, 5 mg/mL, Hameln Pharma GMbH, Germany), fentanyl (Fentanyl Kalceks 50 µg/mL, Kalceks AS, Latvia), rocuronium (Esmeron®, Merck Sharp & Dohme AB, Sweden), 25 mg/mL glucose solution (Braun GmbH, Kronberg im Taunus, Germany), noradrenaline (Hospira Nordic, Stockholm, Sweden) were purchased via ApoEx AB, Stockholm, Sweden. Artificial brain ISF, central nervous system (CNS) Ringer solution (145 mM NaCl, 0.6 mM KCl, 1.2 mM CaCl2, 1 mM MgCl2, 0.2 mM ascorbic acid, KH2PO4 and K2HPO4; pH 7.4) filtered by a 0.45 μm filter (Acrodisc® syringe filter 0.45 μm GHP membrane; Pall Corporation, Port Washington, NY, USA), was prepared in-house. Phosphate buffered saline (PBS: 28 mM Na_2_HPO_4_, 5.6 mM NaH_2_HPO_4_ x 2H_2_O, 95 mM NaCl, in MilliQ water, pH 7.4) filtered by a 0.45 μm filter (Acrodisc® syringe filter 0.45 μm GHP membrane; Pall Corporation, Port Washington, NY, USA), was also prepared in-house.

### Animals

Drug-naïve Swedish landrace pigs of both sexes with body weights of 29.8±1.6 kg, and estimated ages of 10 to 12 weeks, were used in the microdialysis study (n_female_=4, n_male_=3). Importantly, at the age of 10-12 weeks, the pigs have not sexually matured and, hence, it is unlikely to detect any sex-differences. Blank plasma from another four drug-naïve pigs of both sexes with body weights of 26.9±0.6 kg was used for determination the free fraction in plasma (f_u,plasma_) in healthy pigs (n_female_=2, n_male_=2). The brain from one drug-naïve male pig was used for determination of a free fraction in brain (f_u,brain_) in a healthy animal.

The study was approved by the Animal Ethics Committee of Uppsala, Sweden (Ethical approval Dnr. 5.8.18-12768/2021), conducted according to regulations of the Swedish Animal Welfare Agency, and in compliance with the European Communities Council Directive of 22 September 2010 (2010/63/EU). The pigs were monitored as intensive care patients by trained personnel throughout the experiment. The study employed the ARRIVE 2.0 guidelines to ensure transparent and comprehensive reporting (2). The study was not randomized or blinded.

The pre-specified hypothesis of the study was that pigs also had the H^+^/OC transporter function intact and that the extent of oxycodone transport differs in different CNS compartments. The overall objective of this study was therefore to investigate oxycodone concentrations over time in blood, brain, LV CSF and lumbar CSF in healthy and endotoxemia conditions. There was no prior information on the neuro-PK of oxycodone in pigs, hence, a pilot study was performed (n=1), and obtained parameters were used for calculation of the anticipated effect size. Minimally required per-group sample size for a two-tailed t-test study was 6, given the probability level (α = 0.05), the anticipated effect size (Cohen’s d = 1.5), and the desired statistical power level (0.8).

### In vivo study

An overview of the study design is presented in Fig. 1. The experiment lasted for eight hours and was divided into four different stages; briefly, i) preparation of the pigs and implantation of microdialysis probes and intrathecal catheter, ii) a 90-minute stabilization period for conditioning of probes and *in vivo* microdialysis recovery, iii) intravenous (i.v.) oxycodone administration between 0 to 300 minutes, with 0 to 120 minutes as a healthy control period, iv) i.v. LPS administration between 120 to 300 minutes.

#### Preparation of the pigs

On the day of experiment, the pigs were transported from the breeder to Uppsala University. First, the pigs were anesthetized by an intramuscular injection of tiletamine (6 mg/kg), zolazepam (6 mg/kg) and xylazine (2.2 mg/kg). To support the body temperature, the pigs were placed on a heating pad, and infusion fluid warmers (Hotline™, ICU medical, USA) were used. To assure pain relief during the upcoming surgery, a loading dose of 100 µg fentanyl was administered i.v. The anesthesia was maintained by a continuous i.v. infusion of a glucose solution (25 mg/mL) containing ketamine (32 mg/kg/h), fentanyl (4 µg/kg/h) and midazolam (0.1-0.4 mg/kg/h), at an approximate rate of 8 mL/kg/h depending on the need. Rocuronium (10 mg/mL, 0.25 mL/kg/h) was administered as an i.v. infusion as a muscle relaxant to counteract shivering. An IntelliVue MP50 patient monitor (Philips, Germany) was used to monitor body temperature, pressure, saturation and electrocardiography (ECG). Monitoring of the body temperature was performed via a pulmonary artery catheter (Thermodilution catheter, Merit Medical Systems Inc., USA) or via the pharynx. Normo-ventilation (paCO_2_ 4.8-5.8 kPa) was achieved by adjusting the tidal volume and was applied to maintain ventilation as close to a healthy physiological condition as possible. Tracheotomy (tracheal tube size 7.0 mm) was performed and the pigs were mechanically ventilated (Maquet Servo-I, Getinge AB, Stockholm). A pulse oximeter probe was placed on the tail for monitoring. An oxygenation target of 10-30 kPa was achieved by adjusting the fraction of inspired oxygen (FiO_2_) and in case of repeated hypoxemia, increasing the positive end-expiratory pressure (PEEP). A urinary catheter (Rüsch® Briliant, silicone, balloon catheter, size 12 Ch, O.D. 4.0 mm, Teleflex Medical, USA) was placed by a surgical incision of the urinary bladder. A balanced electrolyte solution (Ringer-Acetate, Baxter Viaflo) was administered as an i.v. infusion of 10 mL/kg/h for maintenance of a healthy hydration throughout the experiment. Noradrenaline 20 µg/mL was administered if needed, starting at 5 mL/h, to maintain a mean arterial pressure (MAP) above 60 mmHg. A blood gas analyzer (ABL 800 flex, Radiometer) was used to monitor acid-base status, electrolytes, hematocrit (Hct), levels of hemoglobin and glucose.

A small cervical artery was catheterized for blood sampling and blood pressure measurements (Careflow™, Merit Medical Systems Inc., USA). The external jugular vein was catheterized for infusions and blood pressure measurements (multi-lumen central venous catheterization set, blue FlexTip® catheter, CS-15703, Arrow International Inc., Teleflex Inc., USA). An epidural catheter was placed intrathecally in the lumbar region of the spine for the sampling of lumbar CSF (Portex® Epidural catheter, nylon, 3 lateral eyes, 16G, Smiths Medical ASD, Inc. Keene, NH 03431, USA, n=6) and connected to a pull-pump (REGLO ICC Digital Peristaltic Stand-Alone Pump; 2-Channel, 12 Roller). Microdialysis probes, 10 mm custom-made CMA 20 Elite probe, 60-120 mm shaft, molecular cut off 20 kDa (CMA Microdialysis AB, Kista, Sweden), were implanted in the femoral vein (n=7), frontal cortical region of the brain (from now on referred to as brain, n=7) and LV (n=4) to obtain unbound concentrations in blood, brain ISF and LV CSF. Before placement, the probes were prepared according to the manufacturer’s instructions (CMA Microdialysis AB, Sweden), i.e. placed in Eppendorf tubes containing CNS Ringer solution, and perfused with CNS Ringer solution for a minimum of ten minutes. The probes placed in the CNS were implanted through burr holes over the convexity, fixed using bolt/-s and/or a peripheral venous catheter, and bone wax if needed (COVIDIEN™, BW25G). Sonosite M-Turbo ultrasound (Washington, USA) was used to support the correct placement of the LV probe. The probe positions were visually confirmed terminally. When the preparation was finalized, the pigs were placed in a prone body position.

#### Stabilization period

After the pigs were prepared and before oxycodone was administered, a stabilization period of 90 minutes was initiated for conditioning of the probes and to allow for recovery of the tissues. At the start of the stabilization period, perfusion of the microdialysis probes with CNS Ringer solution was initiated, and maintained throughout the experiment, using a CMA 400 Syringe Pump (CMA Microdialysis AB, Kista, Sweden). The Ringer solution was spiked with 44 ng/mL oxycodone-D3 to continuously measure the recovery across the probe membrane *in vivo*, i.e., using the retrodialysis by calibrator approach (3). To confirm that a relatively short 90-minute stabilization period was enough for brain tissue recovery, a microdialysis study was performed according to previously published protocol (4) in one healthy rat, with the exception that the unbound oxycodone concentration measurements started 90 minutes after probe implantation. The aim was to investigate the impact of a shorter than 24-hour recovery period on the probe recoveries and the extent of BBB uptake. The mean probe recoveries were 5.7 % and 59.3 % in brain and blood, respectively, which were within previously reported ranges at 4.8-18.7 and 37.9-87.5 in brain and blood, respectively, measured after a 24-hour recovery period (4). The K_p,uu,brain_ was 6.6, which was slightly higher than the previously reported K_p,uu,brain_ ranging from 2.9 to 6.1 (4).

#### Oxycodone- and lipopolysaccharide administrations

The oxycodone steady-state was initiated by an i.v. loading dose of 0.16 mg/kg oxycodone over two minutes and maintained by an i.v. infusion of 0.05 mg/kg/h oxycodone throughout the 5-hour experiment (Braun volume pump, B. Braun Medical AB, Danderyd, Sweden). The oxycodone doses were based on clinically relevant concentrations with a target steady-state concentration of 60 ng/mL (5). To estimate pig PK parameters, allometric scaling was applied (6, 7), where human PK were obtained from the literature (5) according to:

${CL}_{human}={CL}_{pig}\times({\frac{{BW}_{human}}{{BW}_{pig}})}^{0.75}$ (Eq. S1)

The LPS dose was selected based on a prior study in pigs, where LPS from *Escherichia coli* (O111:B4) was administered intravenously at a rate of 4 µg/kg/h (8). This dosage resulted in low mortality rates and elevated levels of cytokines such as TNF-α and IL-6, along with significant circulatory, respiratory, and metabolic changes during 6 hours after initiation of LPS administration. This LPS dose has previously been used to induce a septic like state in pigs (8-11).

Initially, a pilot study was conducted as described in the main materials and methods section to validate the feasibility of the study, including suitable oxycodone and LPS doses. As the oxycodone concentrations in the blood and CNS compartments were very stable even after initiation of the LPS infusion, the healthy period of 150 minutes was shortened to 120 minutes and considered enough as a control, and by that, the LPS period was extended by 30 minutes for the following pigs (ID 2-7).

After the healthy period of 120 (n=6) or 150 minutes (n=1), the LPS challenge was initiated as an i.v. infusion of 4 µg/kg/h LPS from *Escherichia coli* (O111:B4, Sigma) throughout the experiment for a total of 180 (n=6) or 150 minutes (n=1) using the Braun volume pump.

To characterize the pathological severity, a pSOFA score was used as a numerical system to evaluate organ failure and septic status (12). pSOFA score is based on evaluation of organ functions of three domains (3D), i.e., respiratory (PaO_2_/FiO_2_ ratio), cardiovascular (MAP) and renal (urine output) functions. In our study, the 3D-pSOFA score was calculated at 4-6 different time points for each animal to evaluate the degree of organ failure and contribute to the evaluation of the LPS-induced inflammation model. However, there are recommendations saying that the LPS challenge is not an appropriate model for replicating human sepsis to be aware of (13).

#### Sample collection

Dialysate samples from the perfused probes in blood, brain and LV were collected in pre-weighed polypropylene microvials (AgnTho’s, Lidingö, Sweden) in 10-minute intervals throughout the experiment, using CMA 142 fraction collectors (Harvard Apparatus Inc., Holliston, MA). Lumbar CSF from the intrathecal space was collected in pre-weighed polypropylene microvials in 10-minute intervals using the peristaltic pull-pump with a withdrawal rate of 4 µL/min, and a fraction collector (CMA 470 Refrigerated Fraction Collector, Harvard Apparatus Inc., Holliston, MA). Dialysate and CSF samples were immediately capped and stored at 6˚C until bioanalysis the following day.

Blood was sampled in Vacutest® tubes before the start of the oxycodone infusion and at 5, 25, 55, 105, 145, 175, 225, 265 or 285 minutes, as well as terminally (Sodium heparin 102 I.U., 6 mL, Polyethylene terephthalate, Vacutest Kima, Arzergrande PD, Italy). After collection, the blood samples were centrifuged at 3000 rpm for ten minutes at 4°C. Thereafter, plasma was collected in 1.5 mL Eppendorf tubes (Eppendorf, Hamburg, Germany). After collection, plasma samples were stored at -20˚C pending bioanalysis. At each blood sampling time point, blood was also collected in heparinized syringes for blood gas analysis, to monitor the health of the pig (3 mL, Portex, arterial blood sampling syringe with dry Lithium and Heparin for gases and electrolytes, Smiths medical ASD, Minneapolis, USA).

Terminally, the brain was isolated and regions including the frontal, parietal, and occipital cortices were collected. The tissue was snap-frozen in dry-iced isopentane and stored at -20˚C pending bioanalysis. Upon isolation, the brain was visually examined to confirm the placement of the probes and to ensure no hemorrhages around the probes.

#### In vivo recovery calculation

Retrodialysis by calibrator was performed *in vivo* throughout the experiment to continuously monitor the recovery of each individual probe over time (3). The calibrator used was oxycodone-D3, thereby, allowing for the assumption of equivalent recovery characteristics. The recovery of oxycodone-D3 was calculated as follows.

$Recovery={(C}_{in}-C_{out})/C_{in}$ (Eq. S2)

Where C_in_ is the concentration of oxycodone-D3 in the perfusion solution of each probe, sampled from the perfusion syringes prior to and after the experiment procedure, and C_out_ is the concentration of oxycodone-D3 in each dialysate sample. Hence, the recovery is the fraction of calibrator that was lost across the probe membrane during the probe perfusion. This fraction is assumed to be equal to the fraction of oxycodone entering the probe perfusate from the brain, and hence, can be used to convert oxycodone dialysate concentrations to estimated brain ISF concentrations.

### Oxycodone partition into blood cells

As oxycodone is partitioning into blood cells (BCs) in rats with reported partition ratios between blood and plasma (C_b_/C_p_) of 1.3±0.3 and 1.2±0.1 (4, 14) the C_b_/C_p_ ratio was determined to examine if this phenomenon is preserved in pigs, according to:

$\frac{C_{b}}{C_{p}}=1-Ht+Ht\times\left( \frac{C_{BC}}{C_{p}} \right)$ (Eq. S3)

Where C_b_/C_p_ is the partition to BCs, Hct is the hematocrit at each time point, C_BC_ is the blood cell oxycodone concentration and C_p_ is the plasma oxycodone concentration. The individual C_b_/C_p_ values can be used to convert oxycodone parameters in plasma to those in blood, e.g., concentrations and clearance (CL). BCs were collected at 25, 105, 175, and 265 minutes after the start of oxycodone infusion, in 1.5 mL Eppendorf tubes. After collection, BC samples were stored at -20˚C pending bioanalysis.

### *In vitro* plasma protein binding and brain tissue binding of oxycodone

Equilibrium dialysis was performed to obtain the fraction of unbound oxycodone in plasma (f_u,plasma_) and brain tissue (f_u,brain_) to evaluate the binding of oxycodone to plasma proteins and to brain tissue in pigs. The technique was performed as previously described (15-17). Fresh, i.e., not frozen, plasma was used to obtain healthy f_u,plasma_. To evaluate if binding properties changed after the LPS challenge, f_u,plasma_ was also determined in plasma obtained at the beginning of the experiment (0 minutes) and terminally (300 minutes). To obtain f_u,brain_ in a healthy condition, brain tissue from a separate pig was used. Terminal brain tissue samples from the LPS-treated pigs were used to obtain f_u,brain_ after LPS challenge. The equilibrium dialysis was performed on a Teflon 96-well plate with semipermeable membranes (molecular weight cut off: 12-14 kDa; Model HTD96b, HTDialysis, Gales Ferry, CT, USA). Briefly, undiluted plasma or diluted brain homogenate (1:9 w:v in PBS, pH 7.4) were spiked with oxycodone at concentrations of 100 ng/mL and 1 µM (315 ng/mL), respectively, based on *in vivo* relevant concentrations. The spiked plasma or brain tissue homogenate was dialyzed against equal volumes of PBS for 6 hours at 37°C with orbital shaking at 200 rpm in a MaxQ4450 incubator (Thermo Fisher Scientific, NinoLab, Sweden). After the incubation, plasma or brain homogenate, and PBS were sampled, matrix-matched and stored at -20°C pending bioanalysis. Matrix matching was performed by adding PBS to the plasma and brain tissue homogenate samples, and blank plasma or brain tissue homogenate to the PBS samples (1:1). f_u,plasma_ and f_u,brain_ were calculated as:

$f_{u,plasma}=\frac{C_{u,buffer}}{C_{p}}$ (Eq. S4)

Where C_u,buffer_ is the oxycodone concentration in the PBS compartment and C_p_ is the oxycodone concentration in the plasma compartment.

$f_{u,brain}=\frac{\frac{1}{D}}{\left( \left( \frac{1}{f_{u,D}} \right)-1 \right)+\frac{1}{D}}$ (Eq. S5)

where D is the dilution factor of 10, and f_u,D_ is the fraction of unbound oxycodone in the brain homogenate calculated as:

$f_{u, D}=\frac{C_{u,buffer}}{C_{tissue}}$ (Eq. S6)

Where C_u,buffer_ is the oxycodone concentration in the PBS compartment and C_tissue_ is the oxycodone concentration in the brain homogenate compartment.

In all equilibrium dialysis experiments, before- and after samples were collected from the spiked tissue or plasma for determination of relative recovery and thermostability, which all were within an acceptable range of 80-120%.

### Bioanalysis

Oxycodone and oxycodone-D3 were quantified in the samples using an ultraperformance liquid chromatography-tandem mass spectrometer (UPLC-MS/MS) as previously described (4). In short, oxycodone-D6 was used as an internal standard (IS). The calibration curves in CNS Ringer solution from the microdialysis experiment, plasma or brain homogenate (1:4 in MilliQ, w:v), included 7-8 standard levels between 0.5-250 ng/mL, and 4-5 quality control (QC) levels at 2-220 ng/mL. The calibration curves used for the equilibrium dialysis samples, included standards in plasma:PBS (1:1, v:v) at eight levels between 0.5-175 ng/mL, and brain tissue homogenate:PBS (1:19, w:v) at ten levels between 1-1000 nM.

#### Sample preparation

Sample preparation was performed as previously described (4, 18). Briefly, dialysate samples, CSF samples, standards, QCs and blanks were diluted 1:3 (v:v) in MilliQ water containing the 8-20 ng/mL IS, in polypropylene microvials. Samples were vortexed, centrifuged and loaded onto 96-well plates.

Plasma samples, standards, QCs and blank plasma, were thawed and vortexed before precipitation 1:2 (v:v) in acetonitrile containing 8-20 ng/mL IS. Samples were vortexed, centrifuged, and the supernatant was diluted 1:2 (v:v) in MilliQ water. After vortexing, the samples were placed onto 96-well plates.

BC samples were thawed and diluted 1:2 (v:v) in MilliQ water to improve oxycodone extraction from the BCs. Thereafter, the BC samples were treated as plasma samples.

Brain tissue samples from the *in vivo* experiment were thawed, weighed, and homogenized in MilliQ water at a ratio of 1:4 (w:v) using an ultrasonication probe (Sonics vibra cell, Chemical instruments AB; Sonic materials Inc., Newtown, USA). All brain tissue homogenate samples underwent the same sample preparation as described for plasma samples.

A sample volume of 5 µL was injected onto the UPLC column, an AQUITY UPLC BEH C18-column (1.7 μm, 2.1 × 50 mm) with a VanGuard C18 Pre-Column (Waters Corporation, Milford, Massachusetts, USA).

#### Quantification of oxycodone and oxycodone-D3

Oxycodone and oxycodone-D3 concentrations were analyzed and determined as previously described (4). In short, an Acquity Ultra-Performance Liquid Chromatography instrument coupled to a Xevo TQ-S Micro mass spectrometer (Waters Corporation, Milford, Massachusetts, USA) was used for quantification. The software were MassLynx version 4.2 and TargetLynx (Waters Corporation, Milford, Massachusetts, USA). The parent 🡪 daughter transitions for oxycodone, oxycodone-D3 and oxycodone-D6 were 316.11 → 298.1 m/z, 319.11 → 301.1 m/z, and 322.18 → 304.1 m/z, respectively. The lower limit of quantification (LLOQ) for oxycodone was set to 0.5 ng/mL for all matrices. Samples below LLOQ were excluded from the data analysis. Bioanalytical runs were accepted if the precision was within 15% and the accuracy within 15%, except at the LLOQ where an accuracy of ±20% was accepted. The inter-day accuracy and precision of oxycodone were ≤ 5.2 % in all matrices analyzed multiple times. The accepted IS recovery of standards and QC samples were 80 to 120%. The linearity of the calibration curves was accepted by a coefficient of determination (R^2^) ≥ 0.99.

### Data analysis of PK parameters

Unbound oxycodone concentrations in blood, brain ISF and LV CSF obtained by microdialysis, were estimated using the probe dialysate sample concentration and the probe recovery, as:

$C_{u}=\frac{C_{dialysate}}{Recovery}$ (Eq. S7)

Where C_u_ is the estimated unbound drug concentration at the probe location *in vivo*, C_dialysate_ is the concentration in the dialysate sample, and recovery is the calibrator recovery across the probe membrane (Eq. S2). The mean recoveries in blood, brain and LV were 36.3±11.0 % (n=5), 21.8±6.0 % (n=5) and 33.2±28.1 % (n=3), respectively (Table S3). There were no differences in recovery between blood, brain and LV, which was expected due to the same probe type and membrane length used in the different locations. To avoid loss of information and to compensate for recovery changes over time (Figure S1), a moving average recovery was applied to obtain unbound concentrations over time (3). Thus, a mean recovery of the calibrator from three subsequent dialysate samples centralized around the sample interval was used (3). This was applied for all the probes. In one pig (ID4), the calibrator recovery in all probes was low or negative, yet, with similar oxycodone concentrations in the dialysate samples as the other pigs. For these probes, the moving average recoveries from mean, location specific, recoveries from the other pigs were applied.

The mean unbound concentration at steady-state (C_u,ss_) and the mean total concentration at steady-state (C_tot,ss_) were calculated in the healthy and LPS-treated periods using samples collected from 55 to 120 or 150 minutes, and from 120 or 150 to 300 minutes, respectively, after the start of oxycodone infusion, to ensure that the mean was based on samples collected when plasma steady-state was reached.

As oxycodone is a basic organic cation, it is almost fully ionized at physiological pH. Using the Henderson-Hasselbalch equation (19, 20), and the reported pK_a_ of 9.1 (21) the fraction of ionized oxycodone in the blood was estimated.

Total oxycodone concentrations in blood (C_tot,blood_) were estimated using the total concentration in plasma (C_tot,plasma_) and individual C_b_/C_p_ (Eq. S3) values in the healthy and LPS-treated periods, respectively.

$C_{tot,blood}=C_{tot,plasma}\times\frac{C_{b}}{C_{p}}$ (Eq. S8)

The fraction of unbound oxycodone in blood (f_u,blood_) was estimated as:

$f_{u,blood}=\frac{f_{u,plasma}}{\frac{C_{b}}{C_{p}}}$ (Eq. S9)

To evaluate the extent of drug distribution to the CNS, the unbound partition coefficients in brain (K_p,uu,brain_), lateral ventricle (K_p,uu,LV_) and lumbar CSF (K_p,uu,lumbarCSF_) were calculated according to the following equation.

$K_{p,uu}=\frac{C_{u,CNS,ss}}{C_{u,blood,ss}}$ (Eq. S10)

Where C_u,CNS,ss_ is the mean unbound concentration in the CNS compartment, including brain ISF or LV CSF, at steady-state, and C_u,blood,ss_ is the mean unbound concentration in blood at steady-state. As lumbar CSF was directly sampled, in a clinical settings manner, the K_p,uu_ was calculated using the mean unbound concentration in lumbar CSF (C_u,lumbarCSF,ss_), total concentration in plasma at steady-state (C_tot,plasma,ss_) corrected for plasma protein binding (f_u,plasma_) as:

$K_{p,uu,lumbarCSF}=\frac{C_{lumbarCSF,ss}}{{(C}_{tot,plasma,ss}{\times f}_{u,plasma})}$ (Eq. S11)

For comparison, the relative extent of drug delivery between two CNS sites (site 1 and 2) was calculated as the ratio of the K_p,uu_-values within each pig, as:

$Relative extent=\frac{K_{p,uu,CNS1}}{K_{p,uu,CNS2}}$ (Eq. S12)

The total partition coefficient in brain (K_p,brain_) was calculated using the total concentrations at steady-state.

$K_{p,brain}=\frac{C_{tot,brain,ss}}{C_{tot,blood,ss}}$ (Eq. S13)

Where C_tot,brain,ss_ is the total oxycodone concentration in brain at steady-state, and C_tot,blood,ss_ is the total oxycodone concentration in blood at steady-state, calculated as the mean of the last two plasma samples (265-300 minutes) and the individual C_b_/C_p_ obtained from the LPS period (Eq. S3).

To evaluate the intra-brain distribution *in vivo*, the apparent unbound volume of distribution (V_u,brain_) was estimated as follows.

$V_{u,brain}=\frac{A_{brain}}{C_{u,brain}}$ (Eq. S14)

Where A_brain_ is the total amount of drug in the brain tissue and C_u,brain_ is the unbound concentration in brain ISF. As the brain tissue was sampled terminally, C_u,brain_ was calculated using the three last time points (275-295 minutes). Intra-brain distribution was evaluated by comparison of V_u,brain_ values with physiological volumes in the brain, where a higher V_u,brain_ value than 1 mL/g brain indicates a more extensive brain tissue binding, and/or distribution to cells and subcellular organelles as previously described (22, 23). V_u,brain_ values were inversed to get an indication of the *in vivo* f_u,brain_, i.e., f_u,brain_ $\approx$ 1/ V_u,brain_ (21).

To evaluate the systemic PK parameters of oxycodone in pigs, CL was estimated using total plasma concentration-time profiles.

$CL=\frac{R_{0}}{C_{tot,plasma,ss}}$ (Eq. S15)

Where R_0_ is the oxycodone infusion rate and C_tot,plasma,ss_ is calculated at 55-120 minutes (healthy period) and 120-300 minutes (LPS period), respectively.

### Statistical analysis

GraphPad Prism version 9.0.0 for Windows (GraphPad Software, San Diego, California USA) was used for performance of statistical analysis. The normal Gaussian distribution of the data was confirmed using Shapiro-Wilk’s and/or D'Agostino & Pearson normality tests. Paired or unpaired two tailed t-tests were used for comparisons of the parameters between the healthy and the LPS period, and between methods. Repeated measures two-way ANOVA followed by Šídák’s multiple comparison test were used for comparisons of probe recovery between locations during healthy and LPS periods, and between C_u,ss,LV_ and C_u,ss,lumbarCSF_ during healthy and LPS periods. Mixed-effects analysis with the Geisser-Greenhouse correction followed by Tukey’s multiple comparisons test was used to compare C_tot,brain_ between brain regions, and K_p,uu_ between each probe location in the healthy and the LPS period, respectively. P-values below 0.05 were used to indicate significance. Data are presented as mean ± standard deviation (SD).

## References

1. Hannon JP, Bossone CA, Wade CE. Normal physiological values for conscious pigs used in biomedical research. Lab Anim Sci. 1990;40(3):293-8.DOI.

2. Percie du Sert N, Hurst V, Ahluwalia A, Alam S, Avey MT, Baker M, Browne WJ, Clark A, Cuthill IC, Dirnagl U, Emerson M, Garner P, Holgate ST, Howells DW, Karp NA, Lazic SE, Lidster K, MacCallum CJ, Macleod M, Pearl EJ, Petersen OH, Rawle F, Reynolds P, Rooney K, Sena ES, Silberberg SD, Steckler T, Würbel H. The ARRIVE guidelines 2.0: Updated guidelines for reporting animal research. PLoS biology. 2020;18(7):e3000410.DOI: 10.1371/journal.pbio.3000410.

3. Bouw MR, Hammarlund-Udenaes M. Methodological aspects of the use of a calibrator in in vivo microdialysis-further development of the retrodialysis method. Pharm Res. 1998;15(11):1673-9.DOI: 10.1023/a:1011992125204.

4. Bällgren F, Hammarlund-Udenaes M, Loryan I. Active Uptake of Oxycodone at Both the Blood-Cerebrospinal Fluid Barrier and The Blood-Brain Barrier without Sex Differences: A Rat Microdialysis Study. Pharmaceutical research. 2023;40(11):2715-30.DOI: 10.1007/s11095-023-03583-0.

5. Pöyhiä R, Olkkola KT, Seppälä T, Kalso E. The pharmacokinetics of oxycodone after intravenous injection in adults. Br J Clin Pharmacol. 1991;32(4):516-8.DOI: 10.1111/j.1365-2125.1991.tb03942.x.

6. Huh Y, Smith DE, Feng MR. Interspecies scaling and prediction of human clearance: comparison of small- and macro-molecule drugs. Xenobiotica. 2011;41(11):972-87.DOI: 10.3109/00498254.2011.598582.

7. Yoshimatsu H, Konno Y, Ishii K, Satsukawa M, Yamashita S. Usefulness of minipigs for predicting human pharmacokinetics: Prediction of distribution volume and plasma clearance. Drug Metab Pharmacokinet. 2016;31(1):73-81.DOI: 10.1016/j.dmpk.2015.11.001.

8. Lipcsey M, Larsson A, Eriksson MB, Sjölin J. Inflammatory, coagulatory and circulatory responses to logarithmic increases in the endotoxin dose in the anaesthetised pig. J Endotoxin Res. 2006;12(2):99-112.DOI: 10.1179/096805106x89053.

9. Strandberg G, Larsson A, Lipcsey M, Berglund L, Eriksson M. Analysis of intraosseous samples in endotoxemic shock--an experimental study in the anaesthetised pig. Acta anaesthesiologica Scandinavica. 2014;58(3):337-44.DOI: 10.1111/aas.12274.

10. Lipcsey M, Larsson A, Eriksson MB, Sjölin J. Effect of the administration rate on the biological responses to a fixed dose of endotoxin in the anesthetized pig. Shock. 2008;29(2):173-80.DOI: 10.1097/SHK.0b013e318067dfbc.

11. Lipcsey M, Larsson A, Olovsson M, Sjölin J, Eriksson MB. Early endotoxin-mediated haemostatic and inflammatory responses in the clopidogrel-treated pig. Platelets. 2005;16(7):408-14.DOI: 10.1080/09537100500163168.

12. Rutai A, Zsikai B, Tallósy SP, Érces D, Bizánc L, Juhász L, Poles MZ, Sóki J, Baaity Z, Fejes R, Varga G, Földesi I, Burián K, Szabó A, Boros M, Kaszaki J. A Porcine Sepsis Model With Numerical Scoring for Early Prediction of Severity. Front Med (Lausanne). 2022;9:867796.DOI: 10.3389/fmed.2022.867796.

13. Osuchowski MF, Ayala A, Bahrami S, Bauer M, Boros M, Cavaillon JM, Chaudry IH, Coopersmith CM, Deutschman CS, Drechsler S, Efron P, Frostell C, Fritsch G, Gozdzik W, Hellman J, Huber-Lang M, Inoue S, Knapp S, Kozlov AV, Libert C, Marshall JC, Moldawer LL, Radermacher P, Redl H, Remick DG, Singer M, Thiemermann C, Wang P, Wiersinga WJ, Xiao X, Zingarelli B. Minimum Quality Threshold in Pre-Clinical Sepsis Studies (MQTiPSS): An International Expert Consensus Initiative for Improvement of Animal Modeling in Sepsis. Shock. 2018;50(4):377-80.DOI: 10.1097/shk.0000000000001212.

14. Boström E, Simonsson US, Hammarlund-Udenaes M. In vivo blood-brain barrier transport of oxycodone in the rat: indications for active influx and implications for pharmacokinetics/pharmacodynamics. Drug metabolism and disposition: the biological fate of chemicals. 2006;34(9):1624-31.DOI: 10.1124/dmd.106.009746.

15. Kalvass JC, Maurer TS. Influence of nonspecific brain and plasma binding on CNS exposure: implications for rational drug discovery. Biopharmaceutics & drug disposition. 2002;23(8):327-38.DOI: 10.1002/bdd.325.

16. Wan H, Rehngren M, Giordanetto F, Bergstrom F, Tunek A. High-throughput screening of drug-brain tissue binding and in silico prediction for assessment of central nervous system drug delivery. Journal of medicinal chemistry. 2007;50(19):4606-15.DOI: 10.1021/jm070375w.

17. Gustafsson S, Sehlin D, Lampa E, Hammarlund-Udenaes M, Loryan I. Heterogeneous drug tissue binding in brain regions of rats, Alzheimer's patients and controls: impact on translational drug development. Scientific reports. 2019;9(1):5308.DOI: 10.1038/s41598-019-41828-4.

18. Boström E, Jansson B, Hammarlund-Udenaes M, Simonsson US. The use of liquid chromatography/mass spectrometry for quantitative analysis of oxycodone, oxymorphone and noroxycodone in Ringer solution, rat plasma and rat brain tissue. Rapid communications in mass spectrometry : RCM. 2004;18(21):2565-76.DOI: 10.1002/rcm.1658.

19. Henderson LJ. Concerning the Relationship Between the Strength of Acids and Their Capacity to Preserve Neutrality. American Journal of Physiology-Legacy Content. 1908;21(2):173-9.DOI: 10.1152/ajplegacy.1908.21.2.173.

20. Hasselbalch K. Die Berechnung Der Wasserstoffzahl Des Blutes Aus Der Freien Und Gebundenen Kohlensäure Desselben, Und Die Sauerstoffbindung Des Blutes Als Funktion Der Wasserstoffzahl. : Julius Springer; 1916.

21. Fridén M, Bergström F, Wan H, Rehngren M, Ahlin G, Hammarlund-Udenaes M, Bredberg U. Measurement of unbound drug exposure in brain: modeling of pH partitioning explains diverging results between the brain slice and brain homogenate methods. Drug metabolism and disposition: the biological fate of chemicals. 2011;39(3):353-62.DOI: 10.1124/dmd.110.035998.

22. Wang Y, Welty DF. The simultaneous estimation of the influx and efflux blood-brain barrier permeabilities of gabapentin using a microdialysis-pharmacokinetic approach. Pharmaceutical research. 1996;13(3):398-403.DOI: 10.1023/a:1016092525901.

23. Fridén M, Ljungqvist H, Middleton B, Bredberg U, Hammarlund-Udenaes M. Improved measurement of drug exposure in the brain using drug-specific correction for residual blood. Journal of cerebral blood flow and metabolism : official journal of the International Society of Cerebral Blood Flow and Metabolism. 2010;30(1):150-61.DOI: 10.1038/jcbfm.2009.200.
